# Supplementary figures and images for: Application of the urban exposome framework using drinking water and quality of life indicators: a proof-of-concept study in Limassol, Cyprus
Source: PeerJ. 2019 May 24;7:e6851. doi: 10.7717/peerj.6851 (PMC6536114; doi:10.7717/peerj.6851)

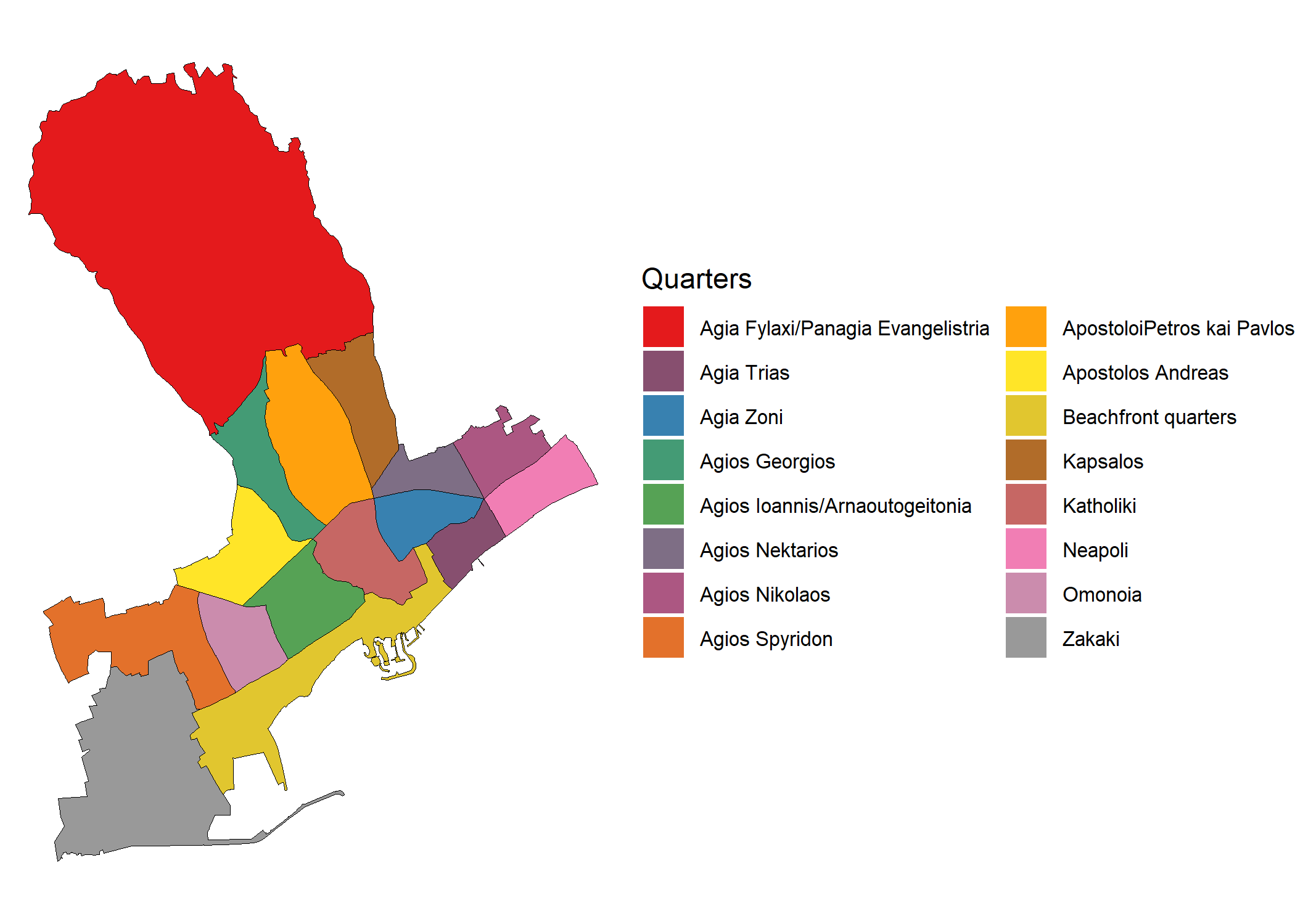

Supplement: Supplemental Information 1 [file peerj-07-6851-s001.png]

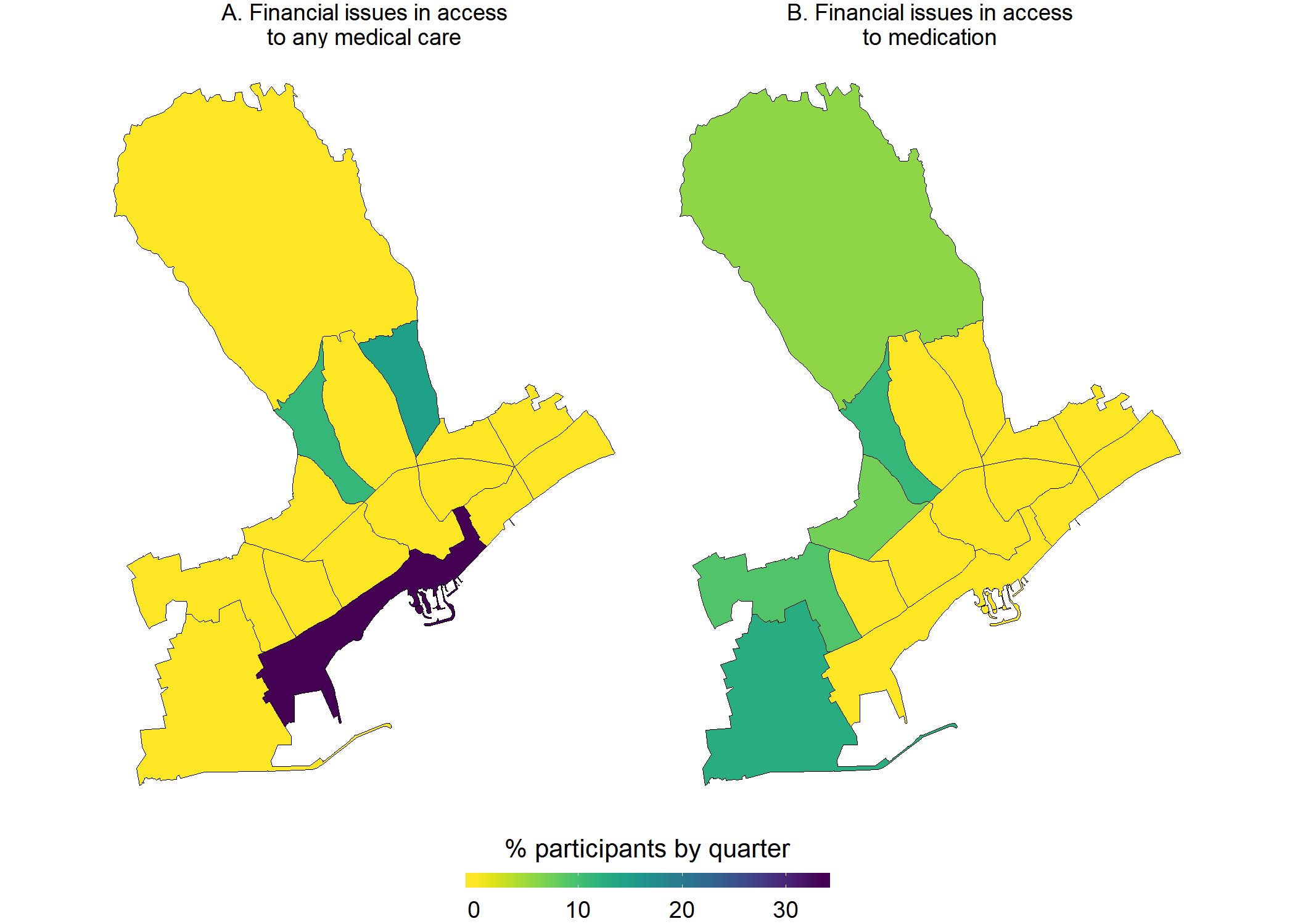

Supplement: Supplemental Information 9 [file peerj-07-6851-s009.zip › SupplementalData_UrbanExposomeWater_PeerJ/Figure4B.png]

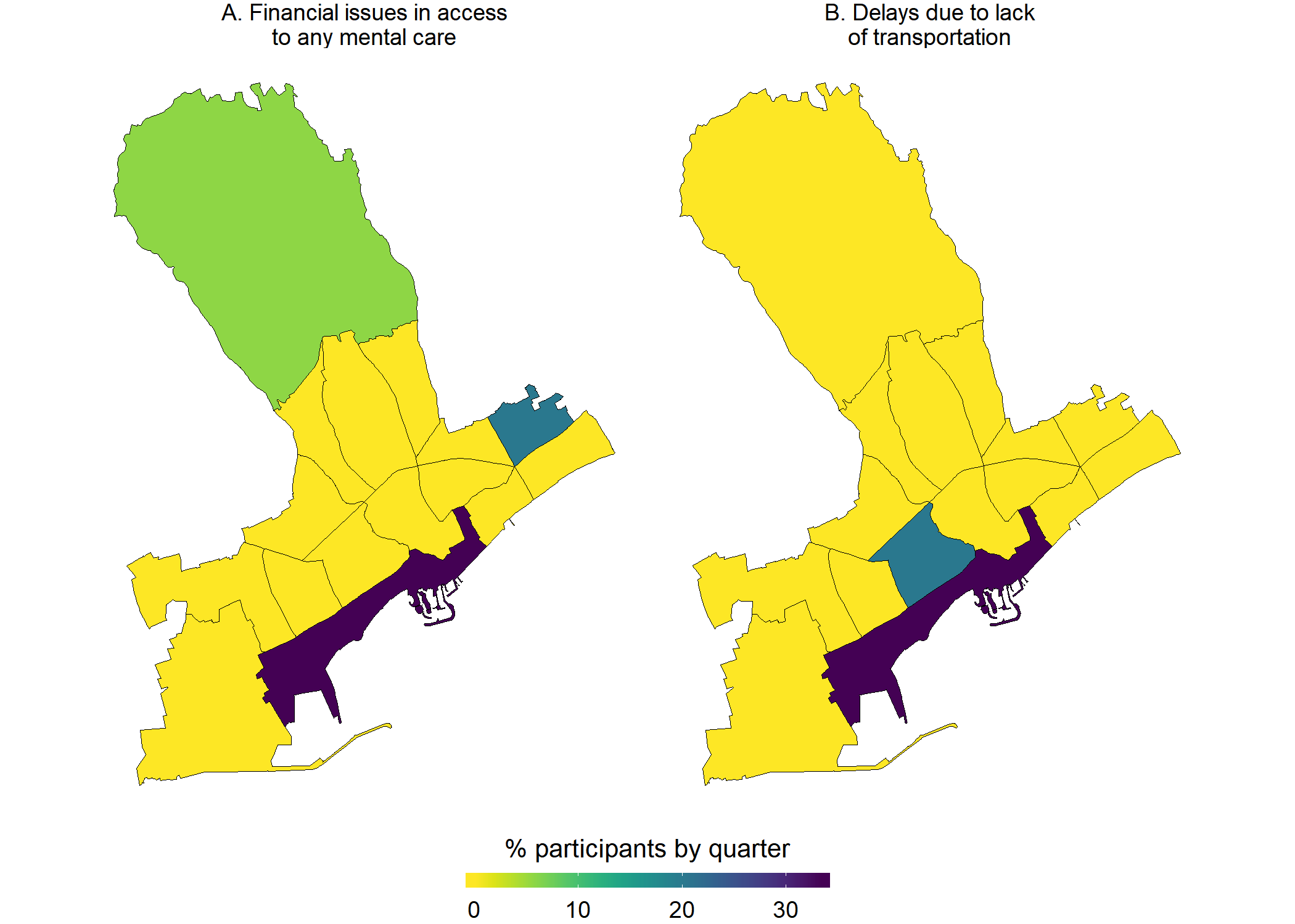

Supplement: Supplemental Information 9 [file peerj-07-6851-s009.zip › SupplementalData_UrbanExposomeWater_PeerJ/Figure4C.png]
