# Supplementary material for: Application of the urban exposome framework using drinking water and quality of life indicators: a proof-of-concept study in Limassol, Cyprus
Source: PeerJ. 2019 May 24;7:e6851. doi: 10.7717/peerj.6851 (PMC6536114; doi:10.7717/peerj.6851)
Supplement: Supplemental Information 2 [file peerj-07-6851-s002.docx]

Microbiological analysis

**Total viable counts at 22 and 37 °C**

Enumeration of heterotrophic bacteria in water was performed by the pour plate method with yeast extract agar (YEA; Oxoid, CM0019, Basingstoke, UK) (Suthar et al. 2009). Briefly, 1 ml of water sample was transferred onto sterile 90-mm Petri dishes, followed by the addition of 15 ml of YEA (previously autoclaved and cooled to 45–50 °C). The contents were mixed by a combination of rapid end-to end shaking and circular movements lasting over a period of 5–10 s. The agar was then allowed to solidify, and incubation of duplicate sets of plates for 3 days at 37 and at 22 °C was practiced.

**Membrane filtration analysis for *E. coli*, coliforms, *Pseudomonas aeruginosa*, and *Enterococus*** **spp.**

The membrane filtration technique was applied for the detection and enumeration of *E. coli*, coliforms, *Pseudomonas aeruginosa*, and *Enterococcus* spp., followed by incubation onto selective media. A volume of 250 ml of the sample was filtered through a 0.45-mm, gridded, sterile membrane, and the filter was then aseptically transferred onto the appropriate agar medium in Petri dish, avoiding air bubbles beneath the membrane. For the *E. coli* and coliform analysis, a cromogenic medium was used (ChromoCult® Coliform Agar, Merck, Darmstadt, Germany), which was able to differentiate between *E. coli* and other coliform bacteria. Plates were incubated inverted at 37 °C for 24 h with red color colonies, indicating the presence of coliform bacteria, whereas the presence of blue colonies indicated the presence of *E. coli* (see Ouattara et al. 2011).

The analysis for *Pseudomonas aeruginosa* was performed onto Pseudomonas Agar (OXOID CM0559, plus Pseudomonas CN selective agar supplement SR0102, Basingstoke, UK). Incubation was set at 25 °C for 2 days with positive colonies of *Pseudomonas aeruginosa* coming up with light green color and fluoresced under UV light at 365 nm. Finally, for enumerating enterococci from the water samples, the membrane filters were incubated onto Slanetz and Bartley medium (OXOID CM0377, Basingstoke, UK) at 44 °C for 4 h and at 37 °C for 2 days. Colonies with red-brown color were enumerated as *Enterococcus* spp. All suspicious colonies were confirmed molecularly via 16S rRNA sequencing as previously described by Botsaris et al. (2015).

**References**

Botsaris, G., Kanetis, L., Slaný, M. et al. Environ Monit Assess (2015) 187: 739.

Ouattara, K. N., Passerat, J., & Servais, P. (2011). Faecal contamination of the water and sediment in the rivers of the Scheldt drainage network. Environmental Monitoring and Assessment, 183(1–4), 243–257.

Suthar, S., Chhimpa, V., & Singh, S. (2009). Bacterial contamination in drinking water: a case study in rural areas of northern Rajasthan, India. Environmental Monitoring and Assessment, 159(1–4), 43–50.
