# Supplementary material for: Application of the urban exposome framework using drinking water and quality of life indicators: a proof-of-concept study in Limassol, Cyprus
Source: PeerJ. 2019 May 24;7:e6851. doi: 10.7717/peerj.6851 (PMC6536114; doi:10.7717/peerj.6851)
Supplement: Supplemental Information 3 [file peerj-07-6851-s003.docx]

|  | **GEO CODE*** | **Area** | **Total**  **(Census 2011**)** | **Estimated sample size** | **Recruited** |  |
| --- | --- | --- | --- | --- | --- | --- |
| Municipality | 5000 | Limassol | 101000 | 120 | 132 |  |
| Quarters | 500017 | Agios Nikolaos | 5631 | 7 | 5 |  |
|  | 500013 | Agios Nektarios | 3397 | 4 | 3 |  |
|  | 500012 | Kapsalos | 6660 | 8 | 7 |  |
|  | 500015 | Agia Trias | 2786 | 3 | 3 |  |
|  | 500016 | Neapoli | 7229 | 9 | 10 |  |
|  | 500008 | Omonoia | 3839 | 5 | 5 |  |
|  | 500014 | Agia Zoni | 4456 | 5 | 5 |  |
|  | 500020 | Agios Spyridon | 9439 | 11 | 11 |  |
|  | 500021 | Zakaki | 5874 | 7 | 8 |  |
|  | 500011 | Apostoloi Petros kai Pavlos | 10412 | 12 | 14 |  |
|  | 500009 | Apostolos Andreas | 9207 | 11 | 14 |  |
|  | 500010 | Agios Georgios | 5060 | 6 | 9 |  |
|  | 500005 | Katholiki | 4647 | 6 | 12 |  |
|  |  |  |  |  |  |  |
| Combined quarters (presented separately and summed together) | 500004 | Agia Napa | 534 | 1 | 1 |  |
|  | 500002 | Tziami Tzentit | 434 | 1 | 1 |  |
|  | 500007 | Tsiflikoudia | 579 | 1 | 1 |  |
|  |  | (Beachfront quarters) | 1547 | 3 | 3 |  |
|  | 500018 | Agia Fylaxis | 14451 | 17 | 17 |  |
|  | 500019 | Panagia Evangelistria | 693 | 1 | 1 |  |
|  |  | (Agia Fylaxis and Panagia Evangelistria) | 15144 | 18 | 18 |  |
|  | 500003 | Arnaoutogeitonia | 905 | 1 | 1 |  |
|  | 500006 | Agios Ioannis | 4767 | 6 | 4 |  |
|  |  | (Arnaoutogeitonia and Agios Ioannis) | 5672 | 7 | 5 |  |
| *Based ont he 2011 Population Census of Cyprus  **Source: Population Census 2011 [Internet]. Statistical Service, Republic of Cyprus. 2014 [cited 2014 Sep 9]. Available from: <http://www.mof.gov.cy/mof/cystat/statistics.nsf/census-2011_cystat_en/census-2011_cystat_en?OpenDocument> | | | | | | |
