# Supplementary material for: Application of the urban exposome framework using drinking water and quality of life indicators: a proof-of-concept study in Limassol, Cyprus
Source: PeerJ. 2019 May 24;7:e6851. doi: 10.7717/peerj.6851 (PMC6536114; doi:10.7717/peerj.6851)
Supplement: Supplemental Information 4 [file peerj-07-6851-s004.docx]

| **Part A** | **Outcomes** |  |  |
| --- | --- | --- | --- |
| Comment on the predictors | Variable | Categories( if applicable) | Score |
| Predictors from blocks/groups 1-6 (number of predictor variables=36) | General health | Very good | 2 |
|  |  | Good | 1 |
|  |  | So and so | 0 |
|  |  | Bad | -1 |
|  |  | Very bad | -2 |
|  |  |  |  |
|  | Chronic disease | Yes | 1 |
|  |  | No | 0 |
|  |  | I don’t know/I don’t want to answer | NA |
| Predictors from blocks/groups 1-5 (number of predictor variables=25) | Any disease past year | Yes | 1 |
|  |  | No | 0 |
|  |  |  |  |
| Part B | Predictors |  |  |
| Block/Group (number of variables) | Description of variables | Categories (if applicable) | Score |
| (B1) Chemical water indicators (n=7) | Trihalomethanes (Total THM, BrTHM, TCM, BDCM, DBCM, TBM; units: μg/L) |  |  |
|  | Free chlorine (units mg/L; ND=0) |  |  |
| (B2) Drinking water habits (n=5) | Number of glasses of water per day by source |  |  |
| (B3) Cleaning activities (n=3) | Mopping, bathroom cleaning, dishwashing (times per week) |  |  |
| (B4) Questionnaire variables (n=5) | Delays in access to health care services due to long waiting lists | I don’t want to answer | NA |
|  |  | No, I didn’t face any delays (reference) | +1 |
|  |  | No, I didn’t need care | 0 |
|  |  | Yes | -1 |
|  | Financial constraints in access to dental care | I don’t want to answer | NA |
|  |  | No, I could afford it (reference) | +1 |
|  |  | No, I didn’t need | 0 |
|  |  | Yes | -1 |
|  | Living close to green space (proximity)  Can do many activities in the green space nearby  There is always someone to help you in the neighborhood | Completely agree (reference) | +2 |
|  |  | Probably agree | +1 |
|  |  | Do not know | 0 |
|  |  | Probably disagree | -1 |
|  |  | Completely disagree | -2 |
| (B5) Participant characteristics (n=5) | Age (years)  BMI (kg/m^2)  Number of cigarettes smoking daily |  |  |
|  | Sex | Female (reference) | 0 |
|  |  | Male | 1 |
| (B6) Self-reported diseases the past year (n=11) | Asthma, respiratory diseases, hypertension, cardiovascular diseases, joint or other muscloskeletal problems, diabetes, allergies, liver disorders, cancer, depression | Yes | 1 |
|  |  | No (reference) | 0 |
|  |  | I don’t know/  I don’t want to answer | NA |
| Notes: NA: excluded as missing | | | |
