# Supplementary material for: Application of the urban exposome framework using drinking water and quality of life indicators: a proof-of-concept study in Limassol, Cyprus
Source: PeerJ. 2019 May 24;7:e6851. doi: 10.7717/peerj.6851 (PMC6536114; doi:10.7717/peerj.6851)
Supplement: Supplemental Information 5 [file peerj-07-6851-s005.docx]

| Packages used in the analysis  [1] mixOmics_6.3.2 MASS_7.3-50 gdtools_0.1.7  [4] bindrcpp_0.2.2 rJava_0.9-10 xlsx_0.6.1  [7] officer_0.3.2 rvg_0.1.9 scales_0.5.0  [10] viridis_0.5.1 viridisLite_0.3.0 broom_0.4.5  [13] reshape2_1.4.3 knitr_1.20 tableone_0.9.3  [16] ISOweek_0.6-2 summarytools_0.8.5 corrplot_0.84  [19] Hmisc_4.1-1 Formula_1.2-3 survival_2.42-3  [22] lattice_0.20-35 forcats_0.3.0 stringr_1.3.1  [25] purrr_0.2.5 readr_1.1.1 tidyr_0.8.1  [28] tibble_1.4.2 tidyverse_1.2.1 lubridate_1.7.4  [31] readxl_1.1.0 RColorBrewer_1.1-2 dplyr_0.7.6  [34] data.table_1.11.4 rgdal_1.3-3 sp_1.3-1  [37] plyr_1.8.4 ggplot2_3.0.0 |
| --- |
