# Supplementary material for: Application of the urban exposome framework using drinking water and quality of life indicators: a proof-of-concept study in Limassol, Cyprus
Source: PeerJ. 2019 May 24;7:e6851. doi: 10.7717/peerj.6851 (PMC6536114; doi:10.7717/peerj.6851)
Supplement: Supplemental Information 6 [file peerj-07-6851-s006.docx]

|  | Overall  (n=132) |
| --- | --- |
| **Years living in Cyprus (%)** |  |
| Less than a year | 3 ( 2.3) |
| 1-5 years | 6 ( 4.5) |
| 6-10 years | 4 ( 3.0) |
| 11-20 years | 3 ( 2.3) |
| More than 20 years | 20 (15.2) |
| All my life | 96 (72.7) |
| **Place of birth (%)** |  |
| Cyprus | 114 (86.4) |
| Other EU country | 13 ( 9.8) |
| Other non-EU country | 5 ( 3.8) |
| **Smoking status (%)** |  |
| I don’t want to answer | 1 ( 0.8) |
| Non-smokers | 81 (61.8) |
| Smokers | 41 (31.3) |
| Occasional smoking | 8 ( 6.1) |
| **Exposure to secondhand smoke (hours per day) (%)** |  |
| I don’t know/I don’t remember | 8 ( 6.2) |
| I don’t want to answer | 1 ( 0.8) |
| Less than 1 hour per day | 35 (27.3) |
| More than 1 hour per day | 32 (25.0) |
| Never or almost never | 52 (40.6) |
| **Alcoholic consumption the past 12 months (%)** |  |
| Daily or almost daily | 11 ( 8.4) |
| 5-6 times per week | 2 ( 1.5) |
| 3-4 times per week | 15 (11.5) |
| 1-2 times per week | 33 (25.2) |
| 2-3 days per month | 23 (17.6) |
| 1 time per month | 8 ( 6.1) |
| Less than 1 time per month | 15 (11.5) |
| Never consumed alcohol in the past 12 months | 7 (5.3) |
| Never or I have consumed alcohol only a few times in my life | 13 (9.9) |
| I don’t want to answer | 4 ( 3.1) |
