# Supplementary material for: Application of the urban exposome framework using drinking water and quality of life indicators: a proof-of-concept study in Limassol, Cyprus
Source: PeerJ. 2019 May 24;7:e6851. doi: 10.7717/peerj.6851 (PMC6536114; doi:10.7717/peerj.6851)
Supplement: Supplemental Information 7 [file peerj-07-6851-s007.docx]

|  |  | Overall (n=132) |
| --- | --- | --- |
|  |  |  |
| **Delays in access to health care services** |  |  |
| Delays in health care due to long waiting list (%) | I don’t want to answer | 6 (4.5) |
|  | No, I didn’t face any delays | 43 (32.6) |
|  | No, I didn’t need care | 68 (51.5) |
|  | Yes | 15 (11.4) |
|  |  |  |
| Delays in health care due to lack of transport (%) | I don’t want to answer | 20 (15.2) |
|  | No, I didn’t face any delays | 43 (32.6) |
|  | No, I didn’t need care | 67 (50.8) |
|  | Yes | 2 (1.5) |
|  |  |  |
| Financial constraints in access to medical care (%) | I don’t want to answer | 9 (6.8) |
|  | No, I could afford it | 45 (34.1) |
|  | No, I didn’t need | 75 (56.8) |
|  | Yes | 3 (2.3) |
|  |  |  |
| **Financial constraints** |  |  |
| Financial constraints in access to dental care (%) | I don’t want to answer | 4 (3.0) |
|  | No, I could afford it | 40 (30.3) |
|  | No, I didn’t need | 70 (53.0) |
|  | Yes | 18 (13.6) |
|  |  |  |
| Financial constraints in access to buy any medications (%) | I don’t want to answer | 6 (4.5) |
|  | No, I could afford it | 45 (34.1) |
|  | No, I didn’t need | 76 (57.6) |
|  | Yes | 5 (3.8) |
|  |  |  |
| Financial constraints in access to mental health care (%) | I don’t want to answer | 11 (8.3) |
|  | No, I could afford it | 5 (3.8) |
|  | No, I didn’t need | 113 (85.6) |
|  | Yes | 3 (2.3) |
|  |  |  |
| **Opinions about green space near the residence** |  |  |
| Enough green spaces (%) | Completely agree | 18 (13.6) |
|  | Probably agree | 29 (22.0) |
|  | Do not know | 8 (6.1) |
|  | Probably disagree | 45 (34.1) |
|  | Completely disagree | 32 (24.2) |
|  |  |  |
| Access to green spaces is easy (%) | Completely agree | 38 (28.8) |
|  | Probably agree | 39 (29.5) |
|  | Do not know | 9 ( 6.8) |
|  | Probably disagree | 24 (18.2) |
|  | Completely disagree | 22 (16.7) |
|  |  |  |
| Living close to green space (proximity) (%) | Completely agree | 45 (34.1) |
|  | Probably agree | 40 (30.3) |
|  | Do not know | 7 ( 5.3) |
|  | Probably disagree | 17 (12.9) |
|  | Completely disagree | 23 (17.4) |
|  |  |  |
| Green spaces nearby are well-maintained (%) | Completely agree | 11 ( 8.3) |
|  | Probably agree | 39 (29.5) |
|  | Do not know | 12 ( 9.1) |
|  | Probably disagree | 39 (29.5) |
|  | Completely disagree | 31 (23.5) |
|  |  |  |
| Relaxing in the green spaces nearby (%) | Completely agree | 14 (10.6) |
|  | Probably agree | 26 (19.7) |
|  | Do not know | 6 ( 4.5) |
|  | Probably disagree | 39 (29.5) |
|  | Completely disagree | 47 (35.6) |
|  |  |  |
| Can do many activities in green space (%) | Completely agree | 8 ( 6.1) |
|  | Probably agree | 18 (13.6) |
|  | Do not know | 9 ( 6.8) |
|  | Probably disagree | 46 (34.8) |
|  | Completely disagree | 51 (38.6) |
| **Opinions about different aspects of life in the neighborhood** |  |  |
| The neighbors are willing to help each other (%) | Completely agree | 41 (31.1) |
|  | Probably agree | 59 (44.7) |
|  | Do not know | 13 ( 9.8) |
|  | Probably disagree | 14 (10.6) |
|  | Completely disagree | 5 ( 3.8) |
|  |  |  |
| Neighbors share the same values (%) | Completely agree | 34 (25.8) |
|  | Probably agree | 57 (43.2) |
|  | Do not know | 23 (17.4) |
|  | Probably disagree | 12 ( 9.1) |
|  | Completely disagree | 6 ( 4.5) |
|  |  |  |
| There is always someone to ask help you(%) | Completely agree | 51 (38.6) |
|  | Probably agree | 56 (42.4) |
|  | Do not know | 15 (11.4) |
|  | Probably disagree | 6 ( 4.5) |
|  | Completely disagree | 4 ( 3.0) |
