# Supplementary material for: Application of the urban exposome framework using drinking water and quality of life indicators: a proof-of-concept study in Limassol, Cyprus
Source: PeerJ. 2019 May 24;7:e6851. doi: 10.7717/peerj.6851 (PMC6536114; doi:10.7717/peerj.6851)
Supplement: Supplemental Information 9 [file peerj-07-6851-s009.zip › SupplementalData_UrbanExposomeWater_PeerJ/Perceptions_UrbanExposome.docx]

Urban exposome - Perceptions study

Chava van der Lek, Xanthi Andrianou

November, 2018

- Number of respondents living in Limassol: 91
- Males: 32 (35.2)%
- Females: 59 (64.8)%
- Mean age: 35.4 years old [range:18-77years old].
- Born in Cyprys 74 (81.3)%
- Born in another EU country: 12 (13.2)%
- Born outside the EU: 5 (5.5)%
- Bachelor’s degree or higher: 76 (83.5)%
- Married: 42 (46.2)%
- Having children: 43 (47.3)%.
- Q31 Out_exp Have you, and to what extent, been exposed OUTSIDE OF YOUR HOUSE (at work, or during your free time) to any of the following conditions in the past 12 months? (1 Severely exposed; 2 Somewhat exposed; 3 Not exposed; 4 Don’t know)

| Exposure | Severely exposed | Somewhat exposed | Not exposed | Don’t know |
| --- | --- | --- | --- | --- |
| Out_exp_noise | 38 (42) | 48 (53) | 5 (5) | 0 (0) |
| Out_exp_air | 40 (44) | 40 (44) | 10 (11) | 1 (1) |
| Out_exp_smell | 15 (16) | 35 (38) | 39 (43) | 2 (2) |
| Out_exp_water | 8 (9) | 22 (24) | 31 (34) | 30 (33) |
| Out_exp_soil | 5 (5) | 15 (16) | 32 (35) | 39 (43) |

- Q32 Worry_chem Are you worried about chemical exposure?
- Q33 Exp_chem Do you think you’re exposed to chemicals on a daily basis
  (1 Yes; 2 No; 3 Maybe)

| - Over | - all |
| --- | --- |
| - n | - 91 |
| - Q32 (%) |  |
| - 1 | - 74 (81.3) |
| - 2 | - 7 ( 7.7) |
| - 3 | - 10 (11.0) |
| - Q33 (%) |  |
| - 1 | - 37 (40.7) |
| - 2 | - 17 (18.7) |
| - 3 | - 37 (40.7) |

- Q35 Water_drink Do you drink tap water?
  (1 Yes; 2 No; 3 Only after cooking it, or using a filter)

| - Over | - all |
| --- | --- |
| - n | - 91 |
| - Q35 (%) |  |
| - 1 | - 26 (28.6) |
| - 2 | - 36 (39.6) |
| - 3 | - 29 (31.9) |

- Q36 Water_concern_1 What is your main concern about the tapwater as drinking water? (max. 1 answer)
  - 1 Chemicals (e.g. heavy metals)
  - 2 Microbes (e.g. bacteria)
  - 3 Taste
  - 5 Other:
  - 4 None

| - - Overall |  |
| --- | --- |
| - - n | - - 91 |
| - - Q36 (%) |  |
| - - 1 | - - 43 (47.3) |
| - - 2 | - - 34 (37.4) |
| - - 3 | - - 9 ( 9.9) |
| - - 4 | - - 1 ( 1.1) |
| - - 5 | - - 4 ( 4.4) |
