# Supplementary material for: Application of the urban exposome framework using drinking water and quality of life indicators: a proof-of-concept study in Limassol, Cyprus
Source: PeerJ. 2019 May 24;7:e6851. doi: 10.7717/peerj.6851 (PMC6536114; doi:10.7717/peerj.6851)
Supplement: Supplemental Information 9 [file peerj-07-6851-s009.zip › SupplementalData_UrbanExposomeWater_PeerJ/Questionnaire1_Perceptions_EN.pdf]

Questionnaire 1 – Perceptions study

Note: the questions used in the analysis of the manuscript “Demonstrating the application of the urban exposome framework using drinking water and quality of life indicators: a proof-of-concept study in Limassol, Cyprus” are highlighted.

Personal information

Q1 What is your gender?

◯ Male

◯ Female

◯ I don't want to answer this question

Q2 What is your age?

Q3 What is the highest education leaving certificate, diploma or education degree you have obtained?

- ◯ Never attended school
- ◯ Not completed primary
- ◯ Primary
- ◯ Gymnasium (3 years)
- ◯ Lyceum (secondary completed)
- ◯ Post Secondary non-tertiary
- ◯ Tertiary level (non-university)
- ◯ University (1st degree)
- ◯ University (Master's degree)
- ◯ University (Doctorate degree)

Q4 Where were you born?

- ◯ Born on Cyprus
- ◯ Born in another EU country:

____

- ◯ Born in a non-EU country

____

Q5 In which of the following areas, did you spend most of childhood (until you turned 18 years old)?

◯ Nicosia (city)

◯ Limassol (city)

◯ Paphos (city)

◯ Larnaca (city)

◯ A village

◯ Not in Cyprys

Q6 What is your current place of residence?

◯ Nicosia (city)

◯ Limassol (city)

◯ Paphos (city)

◯ Larnaca (city)

◯ A village

Display This Question:

If What is your current place of residence? Limassol (city) Is Selected:

Q6B What is your zipcode?

Q7 What is your marital status?

- ◯ Single, never married
- ◯ Cohabiting
- ◯ Married (including registered partnership)
- ◯ Widowed and not remarried
- ◯ Divorced and not remarried

Q8 Do you have children?

- ◯ Yes
- ◯ No

Personal norms and values

Q9 How do perceive your life being controlled? (max. 1 answer)

◯ My life is controlled by me, I decide on what I want to do and achieve in life

◯ My life is controlled by me and my family/friends, I involve others in the decision-making but make the final decision myself

◯ My life is mainly controlled by my family and/or friends

◯ My life is mainly controlled by my family/friends and the government

◯ My life is mainly controlled by the government

Q10 When something bad happens, what do you often see as the cause? (max. 1 answer)

◯ A higher power, for example God, caused it

◯ I have caused it personally

◯ It is a result of other powers in my environment, for example other people

◯ Injustice is the cause, societal inequalities (for example discrimination)

Q11 Whose wellbeing is most important to you? (max. 1 answer)

- ◯ My community
- ◯ My friends
- ◯ My family
- ◯ My own wellbeing

Q12 How do you perceive yourself? (max. 1 answer)

- ◯ As autonomous
- ◯ As a part of a social community
- ◯ As a part of traditions and culture

Q13 How do you consider nature and environment? As basically (max. 1 answer):

○ Stable, but there are certain limits of human induced pressure

○ Good and giving, mother earth provides

○ Flexible, after damage nature will restore itself

○ Overwhelming, the nature makes me feel small

○ Fragile, we are responsible and should thread very lightly

○ Complex, small changes we induce can have big and unforeseen consequences

Q14 According to your own opinion, what terms do reflect your character best? You being (max. 1 answer):

○ Righteous

○ Goodhearted

○ Optimistic

○ Flexible

○ Independent

○ Open minded

Q15 According to your own opinion, what terms do reflect your character best? You being: (max. 1 answer)

- ◯ Conscientious
- ◯ Amiable
- ◯ Ambitious
- ◯ Realistic
- ◯ Autonomous
- ◯ Dynamic

Q16 What do you consider MOST important for a well-functioning society? (max. 1 answer)

- ◯ Truthfulness
- ◯ Kindness
- ◯ Intelligence
- ◯ Adaptability
- ◯ Independence
- ◯ Loyalty

Q17 Do you think a higher social position (for example wealth) could protect people from environmental hazards? (max. 1 answer)

- Definitely yes
- Probably yes
- Might or might not
- Probably not
- Definitely not

Q18 Do you think a lower social position (for example poorness) could increase people's exposure to environmental hazards? (max. 1 answer)

- Definitely yes
- Probably yes
- Might or might not
- Probably not
- Definitely not

Environment

Q19 On what do you think the focus should be in order to tackle environmental problems? (max. 1 answer)

- ◯ Implementing clear rules and regulations
- ◯ Raising awareness and engaging collectively for the cause
- ◯ Developing innovative technologies
- ◯ Cutting down on our consumption patterns
- ◯ Integrate existing knowledge to better understand the complexities of the global environmental problems

Q20 Who do you think has the main responsibility to solve environmental problems? (max. 1 answer)

- ◯ The government
- ◯ Non Governmental Organisations (for example WWF, Greenpeace) or civil actions groups
- ◯ Scientists and inventors
- ◯ I and the lifestylechoices I make, daily
- ◯ All joined forces together
- ◯ We can't help solving (global) environmental problems: they are beyond our influence

Q21 Do you ever worry about the global environment? (max. 1 answer)

- ◯ Yes, very often
- ◯ Yes, sometimes
- ◯ No, almost never
- ◯ No, not at all

Q22 Do you ever worry about Cyprus' environment? (max. 1 answer)

- ◯ Yes, very often
- ◯ Yes, sometimes
- ◯ No, almost never
- ◯ No, not at all

Q23 Do you think you can influence Cyprus' environment with your behavior and actions? (max. 1 answer)

- Definitely yes
- Probably yes
- Might or might not
- Probably not
- Definitely not

Q24 Do you believe in climate change?

- Yes
- No
- Maybe

Display This Question:

If Do you believe in climate change? Yes Is Selected

Q25 How worried are you about climate change on a scale from 1 to 10 (1 as not concerned at all, to 10 as most concerned)

1 2 3 4 5 6 7 8 9 10

Q26 What is, on a global scale, your major environmental worry? (max. 2 answers)

☐ Air pollution

☐ Effects from climate change, such as rising temperatures, extreme weather conditions, droughts or floods

☐ Plastic in the oceans

☐ Water pollution and soil contamination

☐ Rising sea levels

☐ other: ____

Q27 What are your major concerns regarding environmental problems on Cyprus, specifically in relation to industrial pollution? (max. 2 answers)

☐ Air quality

☐ Water quality

☐ Soil contamination/pollution

☐ Chemical exposure

☐ Other: ____

☐ none

Q28 What are your major concerns regarding environmental problems on Cyprus, specifically in relation to agriculture? (max. 2 answers)

☐ Air quality

☐ Water quality

☐ Soil contamination

☐ Chemical exposure

☐ Other: ____

☐ None

Q29 What is your biggest environmental concern where you live? (max. 2 options)

☐ Air quality

☐ Water quality

☐ Soil contamination

☐ Chemical exposure

☐ Waste (waste management, cleanness of public spaces)

☐ Noise

☐ other: ____

☐ None

Q30 Have you, and to what extent, been exposed at HOME to any of the following conditions in the past 12 months?

	Severely exposed	Somewhat exposed	Not exposed	Don't know
Noise (traffic, airplanes, factories, neighbours, animals, restaurants/bars/clubs)	○	○	○	○
Air pollution (fine dust, grime, fume, ozone	○	○	○	○
Bad smells (industry, agriculture, waste)	○	○	○	○
Water pollution (microbes or chemicals in drinking water)	○	○	○	○
Soil contamination (for example a chemical waste dump)	○	○	○	○

Q31 Have you, and to what extent, been exposed OUTSIDE OF YOUR HOUSE (at work, or during your free time) to any of the following conditions in the past 12 months?

	Severely exposed	Somewhat exposed	Not exposed	Don't know
Noise (traffic, airplanes, factories, neighbours, animals, restaurants/bars/clubs)	○	○	○	○
Air pollution (fine dust, grime, fume, ozone)	○	○	○	○
Bad smells (industry, agriculture, waste)	○	○	○	○
Water pollution (microbes or chemicals in drinking water)	○	○	○	○
Soil contamination (for example a chemical waste dump)	○	○	○	○

Q32 Are you worried about chemical exposure?

○ Yes

○ No

○ Maybe

Q33 Do you think you're exposed to chemicals on a daily basis?

- Yes
- No
- Maybe

Q34 Do you think the tap water is safe to drink?

- Yes
- No
- Maybe

Q35 Do you drink tap water?

- Yes
- No
- Only after cooking it, or using a filter

Q36 What is your main concern about the tapwater as drinking water? (max. 1 answer)

- ○ Chemicals (e.g. heavy metals)
- ○ Microbes (e.g. bacteria)
- ○ Taste
- ○ Other: ____
- ○ None

Q37 Where do you get your information about the environment and the place you live? (max. 2 answers)

- ☐ Newspapers
- ☐ Family or friends
- ☐ TV
- ☐ Internet
- ☐ Other: ____
- ☐ None

Q38 Would you like to get more information about environmental hazards in your daily life?

- ◯ Yes
- ◯ No
- ◯ Maybe

Health

Q39 How would you rate your health, on a scale from 1 to 10? (10 as excellent)

1 2 3 4 5 6 7 8 9 10

Q40 Are you worried about your health?

- ◯ Yes, very much
- ◯ Yes, a bit
- ◯ No, not really
- ◯ No, not at all

Q41 How do you think living in a city affects your health?

◯ Positively

◯ Negatively

◯ No influence

Q42 Which environment is healthier, and why? Please provide one or two reasons that you consider make the environment of the city or the villages more healthy

◯ City, because (max. 2 reasons)

____

◯ Villages/rural areas, because (max. 2 reasons)

____

◯ No difference

Q43 Would you like to change your city to make it a healthier environment? Why?

◯ Yes

◯ No

Display This Question:

If Would you like to change your city to make it a healthier environment? Why? Yes Is Selected

Q43.2 If yes, what would you like to change?

Thank you for participating in our research! **Please press the orange button** below to submit your answers. You can write any comments you have before submitting your answers below.
