# Supplementary material for: Application of the urban exposome framework using drinking water and quality of life indicators: a proof-of-concept study in Limassol, Cyprus
Source: PeerJ. 2019 May 24;7:e6851. doi: 10.7717/peerj.6851 (PMC6536114; doi:10.7717/peerj.6851)
Supplement: Supplemental Information 9 [file peerj-07-6851-s009.zip › SupplementalData_UrbanExposomeWater_PeerJ/Questionnaire2_UrbanStudy_GR_ENnotes.pdf]

Questionnaire 2 – Urban population

Note: The questionnaire is available in Greek and the questions used in the analysis of the manuscript “Demonstrating the application of the urban exposome framework using drinking water and quality of life indicators: a proof-of-concept study in Limassol, Cyprus” are highlighted; brief notes on the content of the used questions have been included in English.

Ερωτηματολόγιο

**Ημερομηνία συμπλήρωσης**		**Κωδικός (συμπληρώνεται από τους ερευνητές)**	

Γενικές ερωτήσεις

1. Ταχυδρομικός κώδικας κατοικίας

2. Δήμος/Κοινότητα ____________________

3. Ενορία (αν γνωρίζετε) ____________________

4. Πόσα χρόνια μένετε στην Κύπρο; (years living in Cyprus)

- ❑ Όλη μου τη ζωή
- ❑ Λιγότερο από 1 χρόνο
- ❑ 1-5 χρόνια
- ❑ 6-10 χρόνια
- ❑ 11-20 χρόνια
- ❑ Περισσότερο από 20 χρόνια

5. Γεννηθήκατε στην …

- ❑ Κύπρο
- ❑ Σε άλλη χώρα της Ευρωπαϊκής 'Ενωσης

  Σημειώστε σε ποια άλλη χώρα της Ευρωπαϊκής Ένωσης έχετε γεννηθεί ____________________

- ❑ Σε άλλη χώρα εκτός Ευρωπαϊκής Ένωσης

  Σημειώστε σε ποια άλλη χώρα εκτός της Ευρωπαϊκής Ένωσης έχετε γεννηθεί

  ____

Οικογενειακή κατάσταση και εκπαίδευση

1. **Ποια είναι η οικογενειακή σας κατάσταση; (marital status)**

- ❑ 'Αγαμος/η
- ❑ 'Ατομο που συζεί (σύντροφος)
- ❑ Παντρεμένος/η
- ❑ Χήρος/α που δεν ξαναπαντρέυτηκε
- ❑ Διαζευγμένος/η που δεν ξαναπαντρεύτηκε

2. **Ποιο είναι το υψηλότερο επίπεδο εκπαίδευσης που έχετε ολοκληρώσει με επιτυχία; (education)** *Παρακαλώ περιλάβετε οποιαδήποτε εκπαίδευση πήρατε στα πλαίσια της εργασίας σας.*

- ❑ Δεν έχω φοιτήσει ποτέ σε σχολείο
- ❑ Δεν έχω τελειώσει το δημοτικό
- ❑ Δημοτικό σχολείο
- ❑ Γυμνάσιο (3 χρόνια)
- ❑ Λύκειο/Τεχνική σχολή (απολυτήριο)
- ❑ Μεταλυκειακή εκπαίδευση μη τριτοβάθμια
- ❑ Τριτοβάθμια μη Πανεπιστημιακή
- ❑ Πανεπιστήμιο (πρώτο πτυχίο)
- ❑ Πανεπιστήμιο-Μεταπτυχιακό (μόνο Master's degree)
- ❑ Διδακτορικό

Ερωτήσεις σχετικά με την επαγγελματική σας κατάσταση

1. **Ποια είναι η επαγγελματική σας κατάσταση, αυτή την περίοδο;**

- ❑ Εργαζόμενος/η πλήρους απασχόλησης
- ❑ Εργαζόμενος/η μερικής απασχόλησης
- ❑ Είμαι άνεργος/η και δεν έχω εργαστεί ποτέ
- ❑ Είμαι άνεργος/η, αλλά εργαζόμουν προηγουμένως
- ❑ Οικιακά/φροντίδα παιδιών (χωρίς μισθό)
- ❑ Δεν εργάζομαι για λόγους υγείας
- ❑ Δεν εργάζομαι διότι έχω συνταξιοδοτηθεί
- ❑ Είμαι μαθητής/φοιτητής
- ❑ Υπηρετώ τη στρατιωτική μου θητεία
- ❑ Άλλο (παρακαλώ συμπληρώστε) ______________________________

2. Αν εργάζεστε, ποια είναι η θέση σας στην επιχείρηση/υπηρεσία ή στον οργανισμό όπου εργάζεστε;

- ❑ Αυτοεργοδοτούμενος με υπαλλήλους
- ❑ Αυτοεργοδοτούμενος χωρίς υπαλλήλους
- ❑ Υπάλληλος σε μόνιμη θέση ή σύμβαση εργασίας αορίστου χρόνου
- ❑ Υπάλληλος σε προσωρινή θέση ή σύμβαση εργασίας ορισμένου χρόνου
- ❑ Υπάλληλος σε οικογενειακή επιχείρηση χωρίς μισθό

3. Αν εργάζεστε, σε ποιο τομέα εργάζεστε;

- ❑ Αγροτικές ή/και γεωργικές εργασίες
- ❑ Μεσιτικά
- ❑ Στο δημόσιο τομέα (κυβέρνηση) ή στην άμυνα (στρατός)
- ❑ Εκπαίδευση
- ❑ Σε εργοστάσιο
- ❑ Στον τομέα της υγείας
- ❑ Σε υπηρεσίες δημόσιου συμφέροντος (π.χ. ΑΗΚ, υδατοπρομήθεια)
- ❑ Κατασκευές
- ❑ Πωλήσεις
- ❑ Τουρισμός (ξενοδοχεία και χώροι εστίασης)
- ❑ Στον τομέα της επικοινωνίας
- ❑ Άλλο: ____________________

Ερωτηματολόγιο

Ερωτήσεις σχετικά με την προσωπική αντίληψη της κατάστασης υγείας

1. Πώς είναι γενικά η υγεία σας; Είναι... (general health)

- ❑ Πολύ καλή
- ❑ Καλή
- ❑ Μέτρια
- ❑ Κακή
- ❑ Πολύ κακή
- ❑ Δεν γνωρίζω/δεν είμαι σίγουρος/δεν θυμάμαι
- ❑ Δεν απαντώ

2. Έχετε κάποια μακροχρόνια ασθένεια ή χρόνιο πρόβλημα υγείας (chronic disease)

- ❑ Ναι
- ❑ Όχι
- ❑ Δεν γνωρίζω/δεν είμαι σίγουρος/δεν θυμάμαι
- ❑ Δεν απαντώ

3. Κατά τους τελευταίους 12 μήνες, είχατε κάποια από τις παρακάτω ασθένειες; Σημειώστε στο αντίστοιχο κουτάκι. (diseases the past 12 months)

	Ναι	Όχι	Δεν γνωρίζω	Δεν απαντώ
**Άσθμα (περιλαμβάνεται το αλλεργικό άσθμα)**	☐	☐	☐	☐
**Χρόνια βρογχίτιδα, χρόνια αποφρακτική πνευμονοπάθεια, Εμφύσημα**	☐	☐	☐	☐
**Ψηλή αρτηριακή πίεση - Υπέρταση**	☐	☐	☐	☐
**Καρδιαγγειακές παθήσεις (π.χ. έμφραγμα της καρδιάς (καρδιακή προσβολή) ή χρόνιες επιπτώσεις παλαιού εμφράγματος, στεφανιαία νόσος ή στηθάγχη)**	☐	☐	☐	☐
**Πάθηση των αρθρώσεων (εξαιρείται η αρθρίτιδα)**	☐	☐	☐	☐
**Πάθηση στη μέση ή άλλα χρόνια προβλήματα μέσης (οσφυαλγία-δισκοπάθεια)**	☐	☐	☐	☐
**Πάθηση του αυχένα ή άλλα χρόνια προβλήματα του αυχένα**	☐	☐	☐	☐
**Διαβήτης (εξαιρείται ο διαβήτης της εγκυμοσύνης)**	☐	☐	☐	☐
**Αλλεργία (π.χ. ρινίτιδα, φλεγμονή ματιών, δερματίτιδα, τροφική αλλεργία, κλπ). Εξαιρείται το αλλεργικό άσθμα.**	☐	☐	☐	☐
**Παθήσεις στο συκώτι (π.χ. κίρρωση ήπατος)**	☐	☐	☐	☐
**Καρκίνος (κακοήθης όγκος, λευχαιμία, λέμφωμα)**	☐	☐	☐	☐
**Κατάθλιψη**	☐	☐	☐	☐

4. Χρήση υπηρεσιών υγείας

4.1. Κατά τη διάρκεια των τελευταίων 12 μηνών, αντιμετωπίσατε καθυστέρηση στην παροχή ιατρικής φροντίδας λόγω ………………….. ; (delays in access to health care)

Σημειώστε στο αντίστοιχο κουτάκι στον παρακάτω πίνακα

Λόγοι καθυστέρηση στην παροχή ιατρικής φροντίδας	Ναι	Όχι, δεν αντιμετώπισα καθυστέρηση	Όχι, δεν χρειάστηκα ιατρική φροντίδα	Δεν γνωρίζω	Δεν απαντώ
**Μεγάλης λίστας αναμονής;**	☐	☐	☐	☐	☐
**Απόστασης ή έλλειψης μεταφορικού;**	☐	☐	☐	☐	☐

4.2. Κατά τη διάρκεια των τελευταίων 12 μηνών, χρειαστήκατε κάποιο από τα ακόλουθα είδη φροντίδας, αλλά δεν είχατε την οικονομική ευχέρεια να το έχετε; (financial issues in access to health care)

Σημειώστε στο αντίστοιχο κουτάκι στον παρακάτω πίνακα

Είδη φροντίδας	Ναι	Όχι, είχα οικονομική ευχέρεια	Όχι, δεν χρειάστηκα	Δεν γνωρίζω	Δεν απαντώ
**Ιατρική φροντίδα**	☐	☐	☐	☐	☐
**Οδοντιατρική φροντίδα**	☐	☐	☐	☐	☐
**Συνταγογραφημένα φάρμακα**	☐	☐	☐	☐	☐
**Φροντίδα για ψυχική υγεία**	☐	☐	☐	☐	☐

Πρόληψη και εμβολιασμοί

1. Έχετε εμβολιαστεί ποτέ κατά της γρίπης;

- ❑ Ναι
  Πότε ήταν η τελευταία φορά που εμβολιαστήκατε κατά της γρίπης;
  (χρονιά) …………
- ❑ Όχι (προχωρήστε στην ερώτηση 2)
- ❑ Δεν γνωρίζω/δεν είμαι σίγουρος/δεν θυμάμαι (προχωρήστε στην ερώτηση 2)
- ❑ Δεν απαντώ ((προχωρήστε στην ερώτηση 2)

2. Πότε ήταν η τελευταία φορά που εξεταστήκατε ή μετρήθηκε κάποιος από τους παρακάτω δείκτες;

	Τους τελευταίους 12 μήνες	1-3 χρόνια πριν	3-5 χρόνια πριν	Παλαιότερα από τα τελευταία 5 χρόνια	Ποτέ	Δεν γνωρίζω	Δεν απαντώ
**Αρτηριακή πίεση**	☐	☐	☐	☐	☐	☐	☐
**Χοληστερίνη**	☐	☐	☐	☐	☐	☐	☐
**Σάκχαρο**	☐	☐	☐	☐	☐	☐	☐

3. Κάνατε ποτέ εξέταση για καρκίνο του παχέος εντέρου (π.χ. κολονοσκόπηση, εξέταση ανίχνευσης κρυφού αίματος στα κόπρανα);

- ❑ Ναι
- ❑ Όχι
- ❑ Δεν γνωρίζω/δεν είμαι σίγουρος/δεν θυμάμαι
- ❑ Δεν απαντώ

Δείκτης μάζας σώματος/έτος γέννησης/φύλο

1. **Πόσο είναι το ύψος σας χωρίς παπούτσια; ....εκατοστά (height)**
2. **Πόσο ζυγίζετε χωρίς ρούχα και παπούτσια; ....κιλά (weight)**
3. **Έτος γέννησης ____________ (year of birth)**
4. **Φύλο (sex)**
   - ❑ 'Ανδρας
   - ❑ Γυναίκα
   - ❑ Δεν θα ήθελα να απαντήσω

Ερωτήσεις για γυναίκες

1. **Κάνατε ποτέ μαστογραφία;**
   - ❑ Ναι
   - ❑ Όχι
   - ❑ Δεν γνωρίζω/δεν είμαι σίγουρος/δεν θυμάμαι
   - ❑ Δεν απαντώ
2. **Κάνατε ποτέ τεστ Παπανικολάου;**
   - ❑ Ναι
   - ❑ Όχι
   - ❑ Δεν γνωρίζω/δεν είμαι σίγουρος/δεν θυμάμαι
   - ❑ Δεν απαντώ
3. **Πότε ήταν η τελευταία φορά που κάνατε τεστ Παπανικολάου**
   - ❑ Εντός των τελευταίων 12 μηνών
   - ❑ 1-2 έτη πριν
   - ❑ 2-3 έτη πριν
   - ❑ Παλαιότερα από τα τελευταία 3 έτη
   - ❑ Δεν γνωρίζω/δεν είμαι σίγουρη/δεν θυμάμαι
   - ❑ Δεν απαντώ

Ερωτηματολόγιο

Σωματική δραστηριότητα και διατροφικές συνήθειες

1. **Κάνετε σωματική άσκηση τουλάχιστον 30 λεπτά (π.χ. γρήγορο περπάτημα,τρέξιμο, αεροβική άσκηση, ποδήλατο) τη μέρα, στον ελεύθερό σας χρόνο;**
   - ❑ Καθημερινά
   - ❑ 4-6 φορές τη εβδομάδα
   - ❑ 2-3 φορές την εβδομάδα
   - ❑ 1 φορά την εβδομάδα
   - ❑ 2-3 φορές το μήνα
   - ❑ Μερικές φορές το χρόνο
   - ❑ Δεν ασκούμαι λόγω προβλημάτων υγείας
   - ❑ Δεν ασκούμαι λόγω προβλημάτων κινητικότητας
   - ❑ Δεν ασκούμαι ποτέ

2. **Πόσο συχνά τρώτε πρωινό (π.χ. δημητριακά με γάλα, σάντουιτς κτλ)**
   - ❑ Περισσότερο από 4 φορές την εβδομάδα
   - ❑ Λιγότερο από 4 φορές την εβδομάδα

3. **Κατά μέσο όρο πόσες μερίδες φρούτων καταναλώνετε τη μέρα;**

   …… μερίδες

   Σημείωση: Μια μερίδα ισοδυναμεί περίπου με: 1 μήλο, 1 αχλάδι, 1 ροδάκινο, 1 πορτοκάλι, 2 μανταρίνια, 1 φέττα καρπούζι/πεπόνι (μερίδα εστιατορίου), 7 μικρές φράουλες, 14 κεράσια, 2 δαμάσκηνα, 2 φορμόζες, 2 ακτινίδια, 3 κουταλιές φρουτοσαλάτα (χωρίς κομπόστο ή ζάχαρη), 1 μικρό ποτήρι (150ml) φρέσκο χυμό φρούτων

4. **Κατά μέσο όρο πόσες μερίδες λαχανικών καταναλώνετε τη μέρα;**

   …… μερίδες

   Σημείωση: Μια μερίδα αντιστοιχεί σε: 2 κομμάτια μπρόκολο, 2 μεγάλα κομμάτια κουνουπίδι, 4 κουταλιές λάχανο/σπανάκι/φασολάκι, 1 μέτρια ντομάτα, 1 αγγουράκι, 1 καρότο, 3 κουταλιές λαχανάκια Βρυξελλών, 3 κουταλιές σαλάτα από φρέσκα λαχανικά, 1 μικρό ποτήρι (150ml) φρέσκο χυμό λαχανικών

Ερωτήσεις σχετικά με τη ζωή στη γειτονιά και την πόλη

1. **Κατά μέσο όρο τους τελευταίους 12 μήνες, πόσο συχνά συναντούσατε την οικογένεια και τους συγγενείς σας;**
   - ❑ Καθημερινά
   - ❑ Κάθε εβδομάδα, αλλά όχι κάθε μέρα
   - ❑ Μερικές φορές το μήνα, αλλά όχι κάθε εβδομάδα
   - ❑ Ποτέ

2. **Πόσο συχνά συναντάτε τους φίλους σας**
   - ❑ Καθημερινά
   - ❑ Κάθε εβδομάδα, αλλά όχι κάθε μέρα
   - ❑ Μερικές φορές το μήνα, αλλά όχι κάθε εβδομάδα
   - ❑ Ποτέ

3. **Πόσο συχνά επικοινωνείτε με τους φίλους σας μέσω τηλεφώνου ή του διαδικτύου (με email, στα μέσα κοινωνικής δικτύωσης π.χ. facebook)**
   - ❑ Καθημερινά
   - ❑ Κάθε εβδομάδα, αλλά όχι κάθε μέρα
   - ❑ Μερικές φορές το μήνα, αλλά όχι κάθε εβδομάδα
   - ❑ Ποτέ

4. **Πόσο πολύ αισθάνεστε ότι είστε μέλος ή ταυτίζεστε με τα παρακάτω μέρη;**

	Πάρα πολύ (1)	2	3	4	Καθόλου (5)	Δε γνωρίζω
**Τη γειτονιά**	☐	☐	☐	☐	☐	☐
**Την περιοχή (περίπου σε απόσταση 15-20 λεπτά από την κύρια κατοικίας σας π.χ. δήμος, ενορία)**	☐	☐	☐	☐	☐	☐
**Την πόλη**	☐	☐	☐	☐	☐	☐

5. **Πόσα χρόνια μένετε στην ίδια γειτονιά;**
   - ❑ 0-6 μήνες
   - ❑ 6-12 μήνες
   - ❑ 1-5 χρόνια
   - ❑ Πάνω από 5 χρόνια

6. Σημειώστε αν συμφωνείτε ή διαφωνείτε με τα παρακάτω (life in the neighborhood)

	Συμφωνώ απόλυτα	Μάλλον συμφωνώ	Μάλλον διαφωνώ	Διαφωνώ απόλυτα	Δεν γνωρίζω
**Οι γείτονες μου είναι διατεθειμένοι να βοηθήσουν ο ένας τον άλλον**	☐	☐	☐	☐	☐
**Οι άνθρωποι στη γειτονιά μου δεν έχουν όλοι τις ίδιες αξίες και ιδανικά**	☐	☐	☐	☐	☐
**Πάντα υπάρχει κάποιος να σε βοηθήσει στην γειτονιά**	☐	☐	☐	☐	☐

Ερωτήσεις σχετικά με το περιβάλλον στο οποίο ζείτε

1. Πόσο μακριά απέχει το σπίτι σας από τον κοντινότερο δρόμο με πολύ κίνηση;

❑ Λιγότερο από 20 μέτρα

❑ 20-50 μέτρα

❑ 50-150 μέτρα

❑ Περισσότερο από 150 μέτρα

2. Σημειώστε την απάντηση που ισχύει στο κατάλληλο κουτάκι

	Συνεχώς (σχεδόν πάντα)	Σπάνια	Ποτέ
**Πόσο συχνά περνάνε αυτοκίνητα μπροστά από το σπίτι σας;**	☐	☐	☐
**Πόσο συχνά περνάνε οχήματα μεγάλου κυβισμού, π.χ. φορτηγά, λεωφορεία**	☐	☐	☐

3. Κατά τους τελευταίους 12 μήνες σε τι βαθμό εκτεθήκατε, στο σπίτι σας από θορύβους, π.χ. από αυτοκίνητα και κίνηση, από τους γείτονες, από ζώα, από δραστηριότητες εργοστασίων, από εστιατόρια ή καφέ;

❑ Δεν εκτέθηκα καθόλου

❑ Εκτέθηκα πολύ λίγο

❑ Εκτέθηκα πάρα πολύ

Οι επόμενες ερωτήσεις αφορούν στους χώρους πρασίνου

1. **Σημειώστε σε τι βαθμό συμφωνείτε ή διαφωνείτε με τις παρακάτω δηλώσεις σχετικά με τους χώρους πρασίνου στη γειτονιά σας (green space)**

	Συμφωνώ απόλυτα	Μάλλον συμφωνώ	Μάλλον διαφωνώ	Διαφωνώ απόλυτα	Δεν γνωρίζω
**Οι χώροι πρασίνου στη γειτονιά μου είναι...**					
επαρκείς	☐	☐	☐	☐	☐
εύκολα προσβάσιμοι	☐	☐	☐	☐	☐
είναι κοντά στο σπίτι μου	☐	☐	☐	☐	☐
καλά διατηρημένοι	☐	☐	☐	☐	☐

	Συμφωνώ απόλυτα	Μάλλον συμφωνώ	Μάλλον διαφωνώ	Διαφωνώ απόλυτα	Δεν γνωρίζω
**Στους χώρους πρασίνου στη γειτονιά μου...**					
μπορώ να πάω και να χαλαρώσω στους χώρους πρασίνου στη γειτονιά μου	☐	☐	☐	☐	☐
μπορώ να κάνω πολλές δραστηριότητες	☐	☐	☐	☐	☐

Ευχαριστούμε πολύ!

Ερωτηματολόγιο

Κάπνισμα και κατανάλωση αλκοόλ

1. Καπνίζετε; (smoking)

- ❑ Ναι, καθημερινά
- ❑ Ναι, περιστασιακά
- ❑ Καθόλου (προχωρήστε στην ερώτηση 2)
- ❑ Δεν γνωρίζω/δεν είμαι σίγουρος/δεν θυμάμαι (προχωρήστε στην ερώτηση 2)
- ❑ Δεν απαντώ (προχωρήστε στην ερώτηση 2)

Εάν απαντήσατε «Ναι» στην προηγούμενη ερώτηση

1.1. Ποιο προϊόν καπνίζετε κυρίως;

- ❑ Τσιγάρα (βιομηχανοποιημένα ή στριφτά)
- ❑ Πούρα
- ❑ Πίπα
- ❑ Άλλα προϊόντα
- ❑ Δεν γνωρίζω/δεν είμαι σίγουρος/δεν θυμάμαι

1.2. Κατά μέσο όρο, πόσα τσιγάρα καπνίζετε καθημερινά; (number of cigarettes smoking per day)

Αριθμός τσιγάρων (βιομηχανοποιημένων ή στριφτών) …………………

- ❑ Δεν γνωρίζω/δεν είμαι σίγουρος/δεν θυμάμαι
- ❑ Δεν απαντώ

2. Πόσο συχνά εκτίθεστε σε καπνό από προϊόντα που καπνίζουν άλλοι σε κλειστό χώρο, π.χ. στο σπίτι, στην εργασία, σε δημόσιους χώρους, σε εστιατόρια και στα μέσα μαζικής μεταφοράς; (secondhand smoking)

- ❑ Ποτέ ή σχεδόν ποτέ
- ❑ Λιγότερο από 1 ώρα ημερησίως
- ❑ Περισσότερο από 1 ώρα ημερησίως
- ❑ Δεν γνωρίζω/δεν είμαι σίγουρος/δεν θυμάμαι
- ❑ Δεν απαντώ

3. Κατά τη διάρκεια των τελευταίων 12 μηνών, πόσο συχνά καταναλώσατε αλκοολούχα ποτά, οποιουδήποτε είδους (μπύρα, κρασί, κονιάκ, ζιβανία, ουίσκι, ούζο, λικέρ, κοκτέιλς με αλκοόλ, breezers ή άλλα); (alcohol consumption)

- ❑ Καθημερινά ή σχεδόν καθημερινά
- ❑ 5-6 μέρες την εβδομάδα
- ❑ 3-4 μέρες την εβδομάδα
- ❑ 1-2 μέρες την εβδομάδα
- ❑ 2-3 μέρες το μήνα
- ❑ 1 φορά το μήνα
- ❑ Λιγότερο από 1 φορά το μήνα
- ❑ Ποτέ τους τελευταίους 12 μήνες, το έκοψα
- ❑ Ποτέ ή μόνο ελάχιστες φορές στη ζωή μου
- ❑ Δεν γνωρίζω/δεν είμαι σίγουρος/δεν θυμάμαι
- ❑ Δεν απαντώ

Ευχαριστούμε πολύ!

Ερωτηματολόγιο

**Ημερομηνία συμπλήρωσης**		**Κωδικός (συμπληρώνεται από τους ερευνητές)**	

Συνήθειες κατανάλωσης νερού και δραστηριοτήτων καθαριότητας

1. Από ποιες πηγές καταναλώνετε νερό; (water sources)

❑ Υδατοπρομήθεια
❑ Εμφιαλωμένο νερό
❑ Νερό από αυτόματους πωλητές (κερματοδέκτες)
❑ Νερό πηγής
❑ Άλλο (παρακαλώ συμπληρώστε) ....

Χρησιμοποιείτε φίλτρο στην κεντρική βρύση του σπιτιού; (use of filter in the main tap)

❑ Ναι ❑ Όχι

2. Πόσα ποτήρια νερό καταναλώνετε τη μέρα από κάθε πηγή (water consumption)

1 ποτήρι νερό αντιστοιχεί περίπου σε ποσότητα 250ml. Σημειώστε τον αριθμό ποτηριών ανά πηγή νερού που χρησιμοποιείτε.

	Αριθμός ποτηριών
**Υδατοπρομήθεια**	
**Εμφιαλωμένο νερό**	
**Νερό από αυτόματους πωλητές**	
**Νερό πηγής**	
**Άλλο (παρακαλώ συμπληρώστε) ______________________**	

3. Πόσες μέρες την εβδομάδα εκτελείτε κατά μέσο όρο της παρακάτω δραστηριότητες; (cleaning activities)

	Μέρες την εβδομάδα
**Πλύσιμο πιάτων**	
**Σφουγγάρισμα χώρων σπιτιού**	
**Καθαρισμός μπάνιου**	

Χρήση πλαστικών δοχείων

1. Χρησιμοποιείτε πλαστικά δοχεία για την αποθήκευση φαγητού;

❑ Ναι (απαντήστε και στην ερώτηση από κάτω)

Κάνετε καθημερινή χρήση των πλαστικών δοχείων (π.χ. για μεταφορά γευμάτων στη δουλειά); ❑ Ναι ❑ Όχι καθημερινά

❑ Όχι

2. Πόσες φορές την εβδομάδα χρησιμοποιείτε πλαστικές συσκευασίες στον φούρνο μικροκυμάτων;

__________ φορές

Χρήση αλατιού

1. **Χρησιμοποιείτε ιωδιούχο αλάτι;**
   - ❑ Ναι, πάντα
   - ❑ Δεν γνωρίζω/δεν προσέχω την ετικέτα
   - ❑ Χρησιμοποιώ υποκατάστατο αλατιού
   - ❑ Γνωρίζω τι είναι το ιωδιούχο αλάτι αλλά δεν χρησιμοποιώ

Χρήση φυτοφαρμάκων και εντομοκτόνων

1. **Χρησιμοποιείτε φυτοφάρμακα στον κήπο, την αυλή, ή στα λουλούδια σας;**
   - ❑ Ναι
   - ❑ Όχι

   Εάν απαντήσατε «Ναι» στην προηγούμενη ερώτηση

   1.1. Πότε ήταν η τελευταία φορά που χρησιμοποιήσατε φυτοφάρμακα;
   Μήνας ………………… Χρονιά ………….
   Ποια ήταν η μάρκα των φυτοφαρμάκων που χρησιμοποιήσατε; ……………………………….

2. **Χρησιμοποιείτε εντομοκτόνα ή εντομοαπωθητικά;**
   - ❑ Ναι, σε χώρους (π.χ. στο σπίτι) και σώματος
   - ❑ Ναι, μόνο σε χώρους
   - ❑ Ναι, μόνο σώματος
   - ❑ Δεν χρησιμοποιώ καθόλου (*συνεχίστε στην επόμενη ενότητα*)
   - ❑ Δεν γνωρίζω/δεν είμαι σίγουρος/δεν θυμάμαι (*συνεχίστε στην επόμενη ενότητα*)

   Εάν απαντήσατε «Ναι» στην προηγούμενη ερώτηση

   2.1. Πότε ήταν η τελευταία φορά που χρησιμοποιήσατε εντομοκτόνα ή εντομοαπωθητικά;
   Μήνας......... Χρονιά .... τελευταίας χρήσης

Κατανάλωση ψαριών

1. **Πόσες φορές την εβδομάδα καταναλώνετε φρέσκα ψάρια; ....** φορές

2. **Πόσες φορές την εβδομάδα καταναλώνετε ψάρια σε κονσέρβες (π.χ. τόνος); ....** φορές

Ερωτηματολόγιο

Κάπνισμα και κατανάλωση αλκοόλ

1. Καπνίζετε; (smoking)

- ❑ Ναι, καθημερινά
- ❑ Ναι, περιστασιακά
- ❑ Καθόλου (προχωρήστε στην ερώτηση 2)
- ❑ Δεν γνωρίζω/δεν είμαι σίγουρος/δεν θυμάμαι (προχωρήστε στην ερώτηση 2)
- ❑ Δεν απαντώ (προχωρήστε στην ερώτηση 2)

Εάν απαντήσατε «Ναι» στην προηγούμενη ερώτηση

1.1. Ποιο προϊόν καπνίζετε κυρίως;

- ❑ Τσιγάρα (βιομηχανοποιημένα ή στριφτά)
- ❑ Πούρα
- ❑ Πίπα
- ❑ Άλλα προϊόντα
- ❑ Δεν γνωρίζω/δεν είμαι σίγουρος/δεν θυμάμαι

1.2. Κατά μέσο όρο, πόσα τσιγάρα καπνίζετε καθημερινά; (number of cigarettes smoking per day)

Αριθμός τσιγάρων (βιομηχανοποιημένων ή στριφτών) …………………

- ❑ Δεν γνωρίζω/δεν είμαι σίγουρος/δεν θυμάμαι
- ❑ Δεν απαντώ

2. Πόσο συχνά εκτίθεστε σε καπνό από προϊόντα που καπνίζουν άλλοι σε κλειστό χώρο, π.χ. στο σπίτι, στην εργασία, σε δημόσιους χώρους, σε εστιατόρια και στα μέσα μαζικής μεταφοράς; (secondhand smoking)

- ❑ Ποτέ ή σχεδόν ποτέ
- ❑ Λιγότερο από 1 ώρα ημερησίως
- ❑ Περισσότερο από 1 ώρα ημερησίως
- ❑ Δεν γνωρίζω/δεν είμαι σίγουρος/δεν θυμάμαι
- ❑ Δεν απαντώ

3. Κατά τη διάρκεια των τελευταίων 12 μηνών, πόσο συχνά καταναλώσατε αλκοολούχα ποτά, οποιουδήποτε είδους (μπύρα, κρασί, κονιάκ, ζιβανία, ουίσκι, ούζο, λικέρ, κοκτέιλς με αλκοόλ, breezers ή άλλα); (alcohol consumption)

- ❑ Καθημερινά ή σχεδόν καθημερινά
- ❑ 5-6 μέρες την εβδομάδα
- ❑ 3-4 μέρες την εβδομάδα
- ❑ 1-2 μέρες την εβδομάδα
- ❑ 2-3 μέρες το μήνα
- ❑ 1 φορά το μήνα
- ❑ Λιγότερο από 1 φορά το μήνα
- ❑ Ποτέ τους τελευταίους 12 μήνες, το έκοψα
- ❑ Ποτέ ή μόνο ελάχιστες φορές στη ζωή μου
- ❑ Δεν γνωρίζω/δεν είμαι σίγουρος/δεν θυμάμαι
- ❑ Δεν απαντώ

Ευχαριστούμε πολύ!
