# Supplementary material for: Application of the urban exposome framework using drinking water and quality of life indicators: a proof-of-concept study in Limassol, Cyprus
Source: PeerJ. 2019 May 24;7:e6851. doi: 10.7717/peerj.6851 (PMC6536114; doi:10.7717/peerj.6851)
Supplement: Supplemental Information 9 [file peerj-07-6851-s009.zip › SupplementalData_UrbanExposomeWater_PeerJ/UrbanExposomeWater_additionalSurvey_graphs_2019-05-03.pptx]

## Slide 1
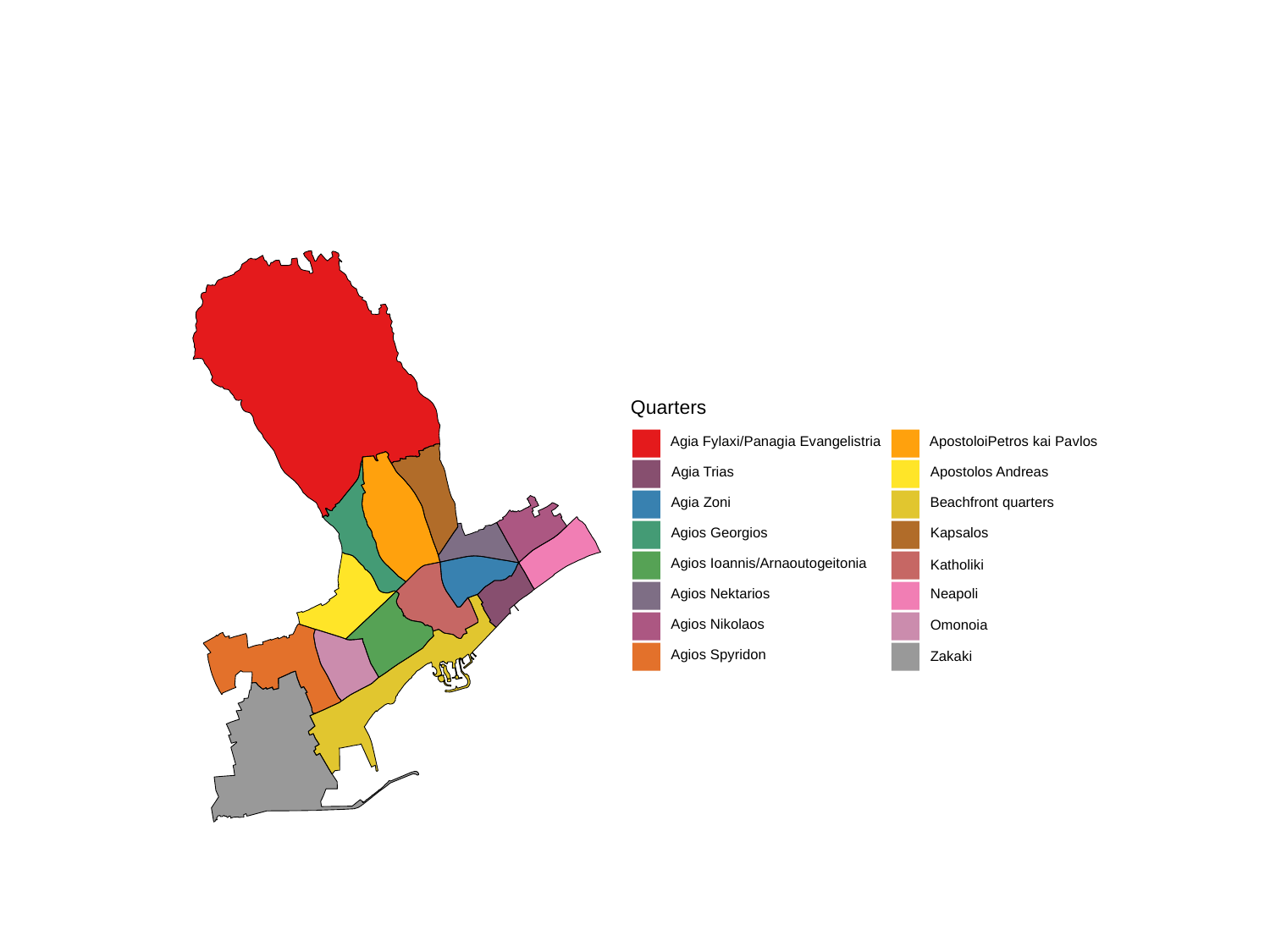

Quarters
Agia Fylaxi/Panagia Evangelistria
ApostoloiPetros kai Pavlos
Agia Trias
Apostolos Andreas
Beachfront quarters
Agia Zoni
Agios Georgios
Kapsalos
Agios Ioannis/Arnaoutogeitonia
Katholiki
Agios Nektarios
Neapoli
Agios Nikolaos
Omonoia
Agios Spyridon
Zakaki

## Slide 2
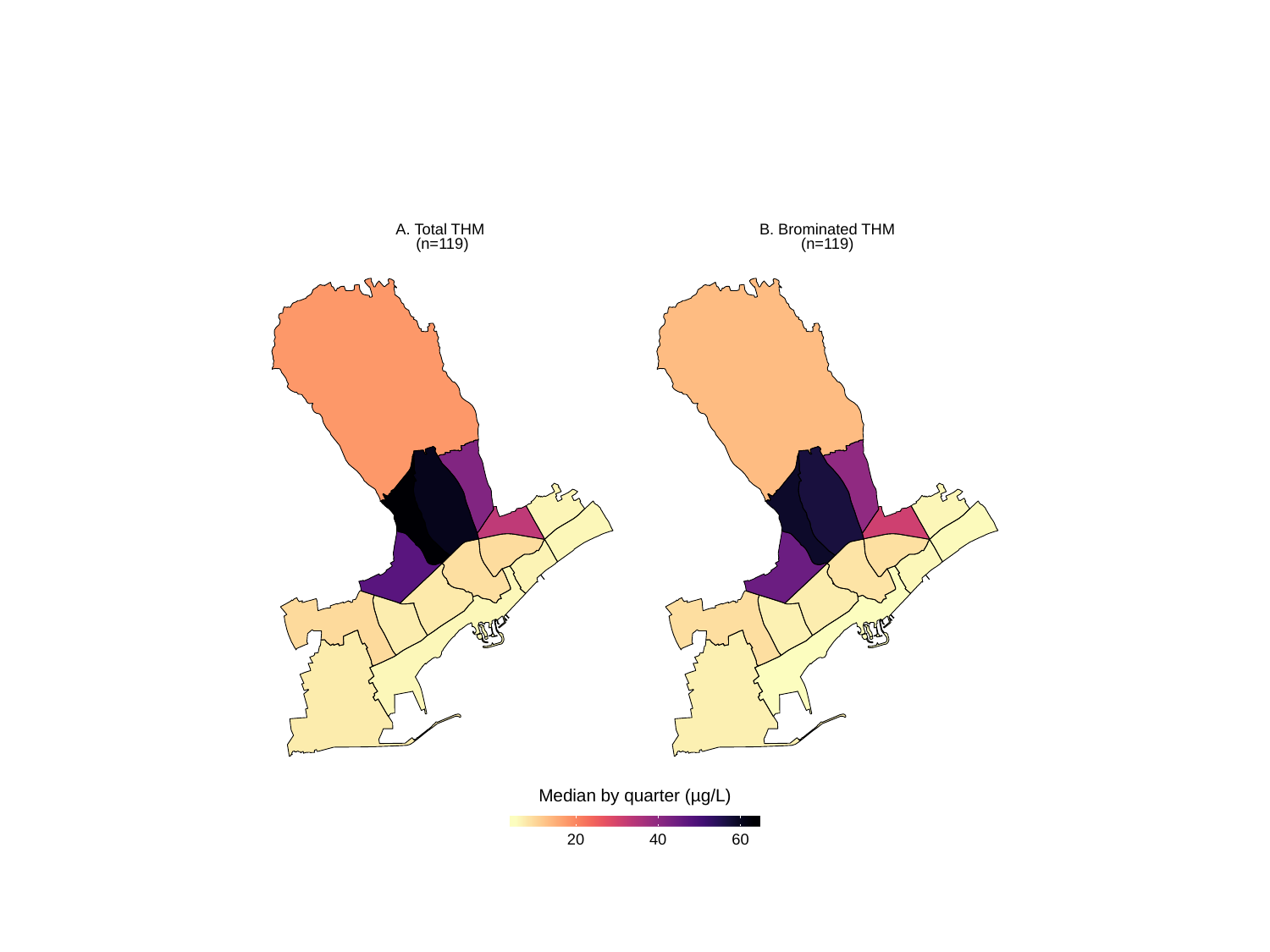

A. Total THM
B. Brominated THM
(n=119)
(n=119)
Median by quarter (µg/L)
20
40
60

## Slide 3
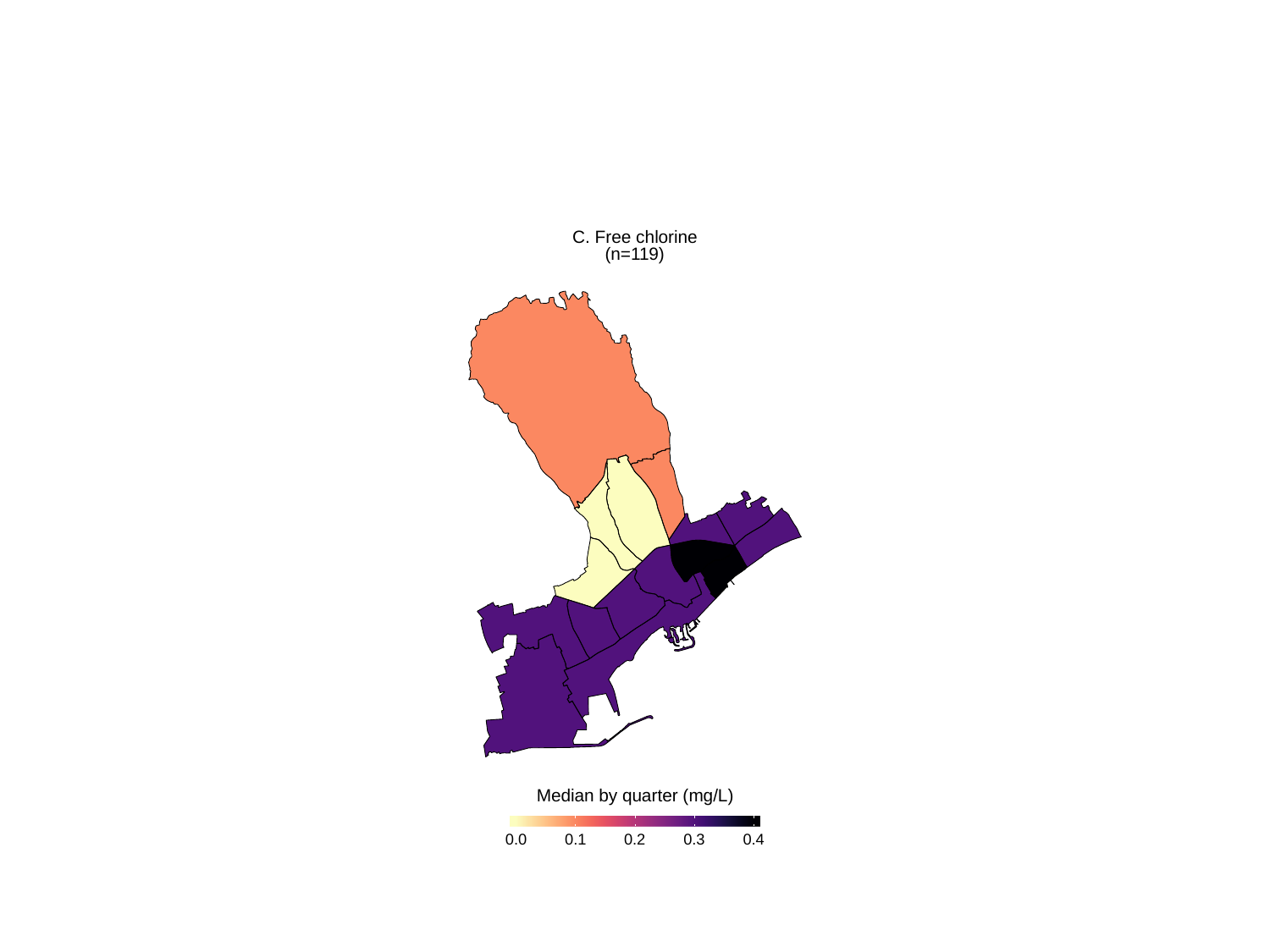

C. Free chlorine
(n=119)
Median by quarter (mg/L)
0.3
0.0
0.1
0.2
0.4

## Slide 4
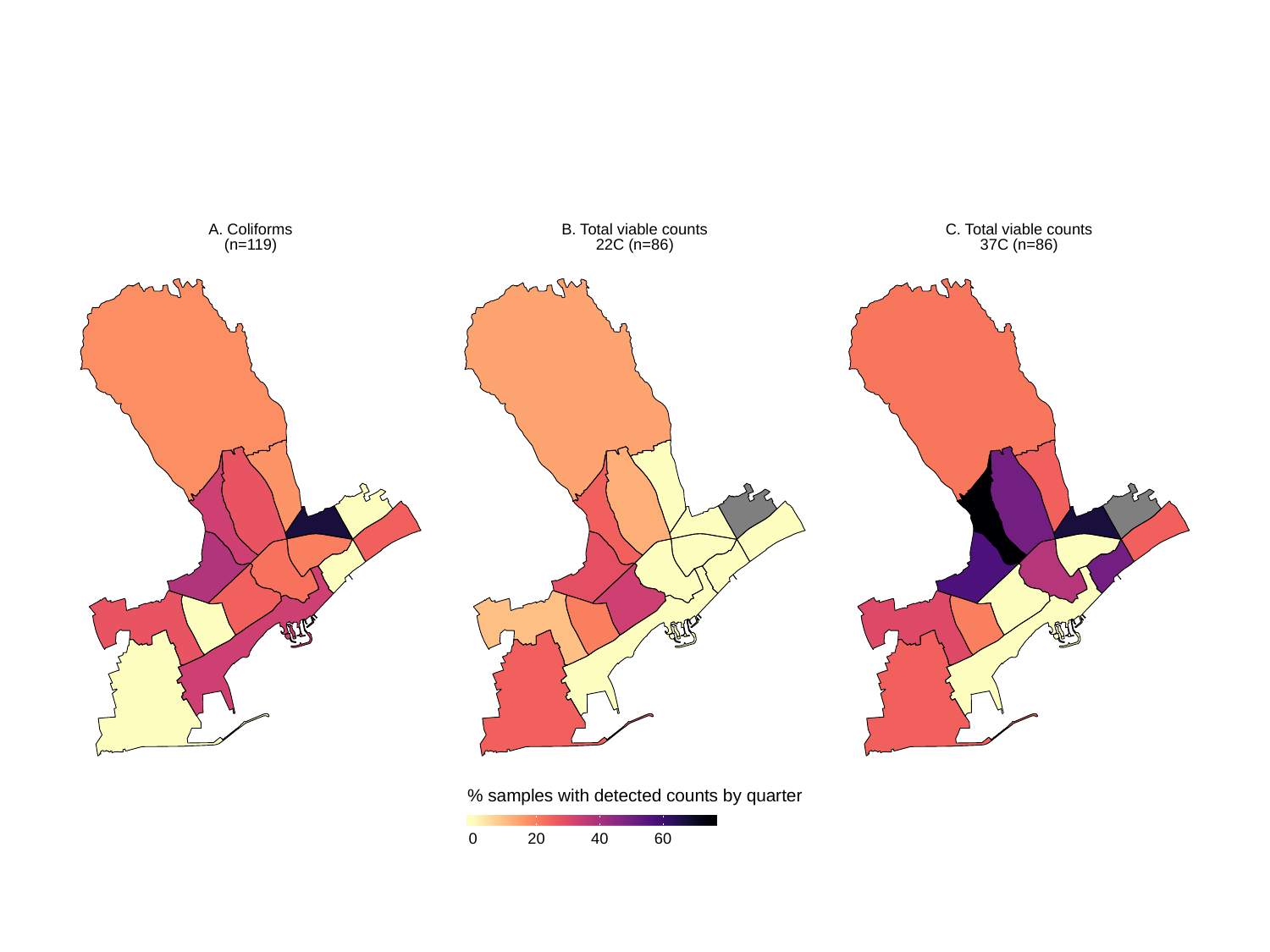

A. Coliforms
C. Total viable counts
B. Total viable counts
(n=119)
22C (n=86)
37C (n=86)
% samples with detected counts by quarter
0
20
40
60

## Slide 5
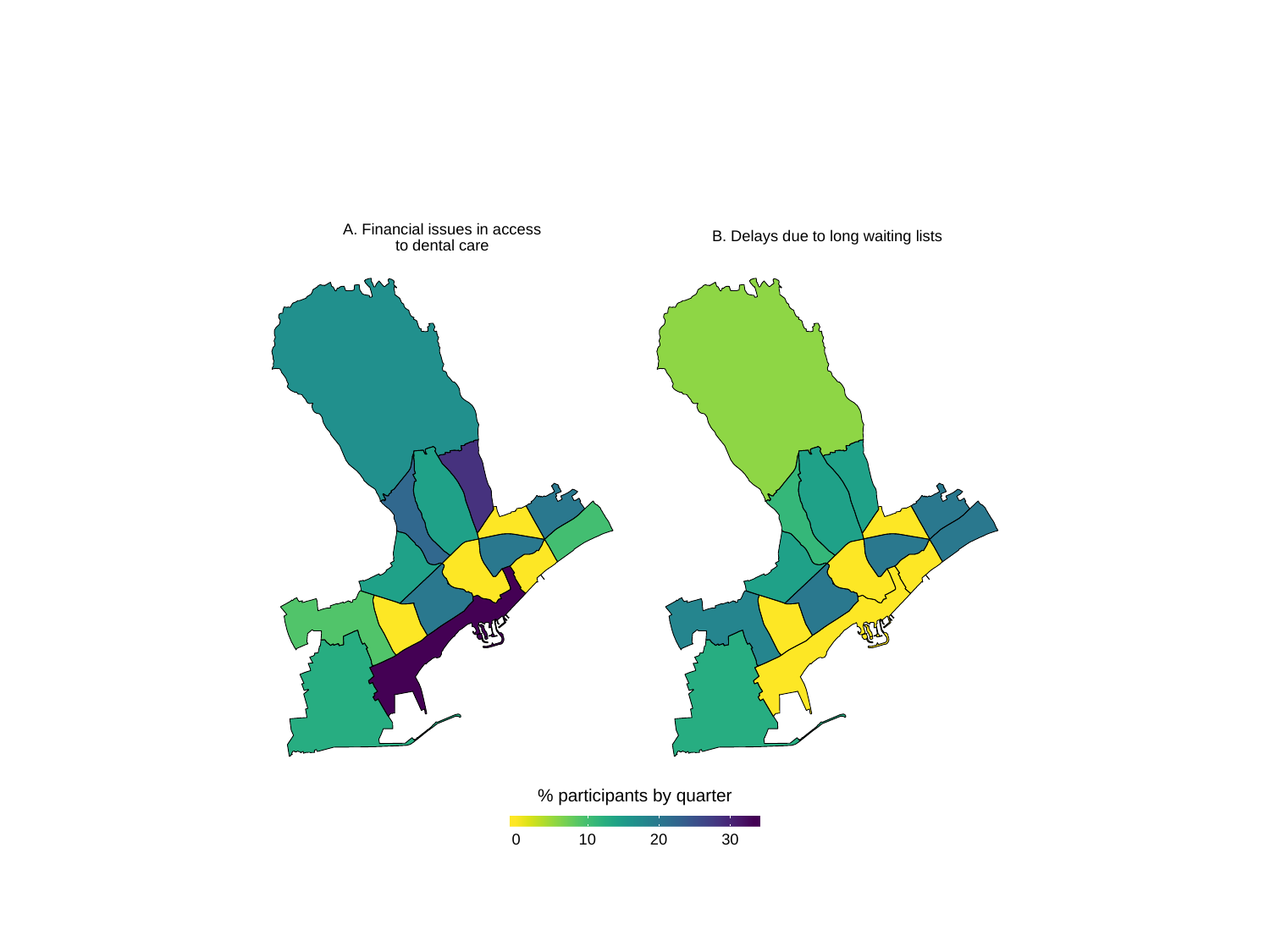

A. Financial issues in access
B. Delays due to long waiting lists
to dental care
% participants by quarter
30
0
10
20

## Slide 6
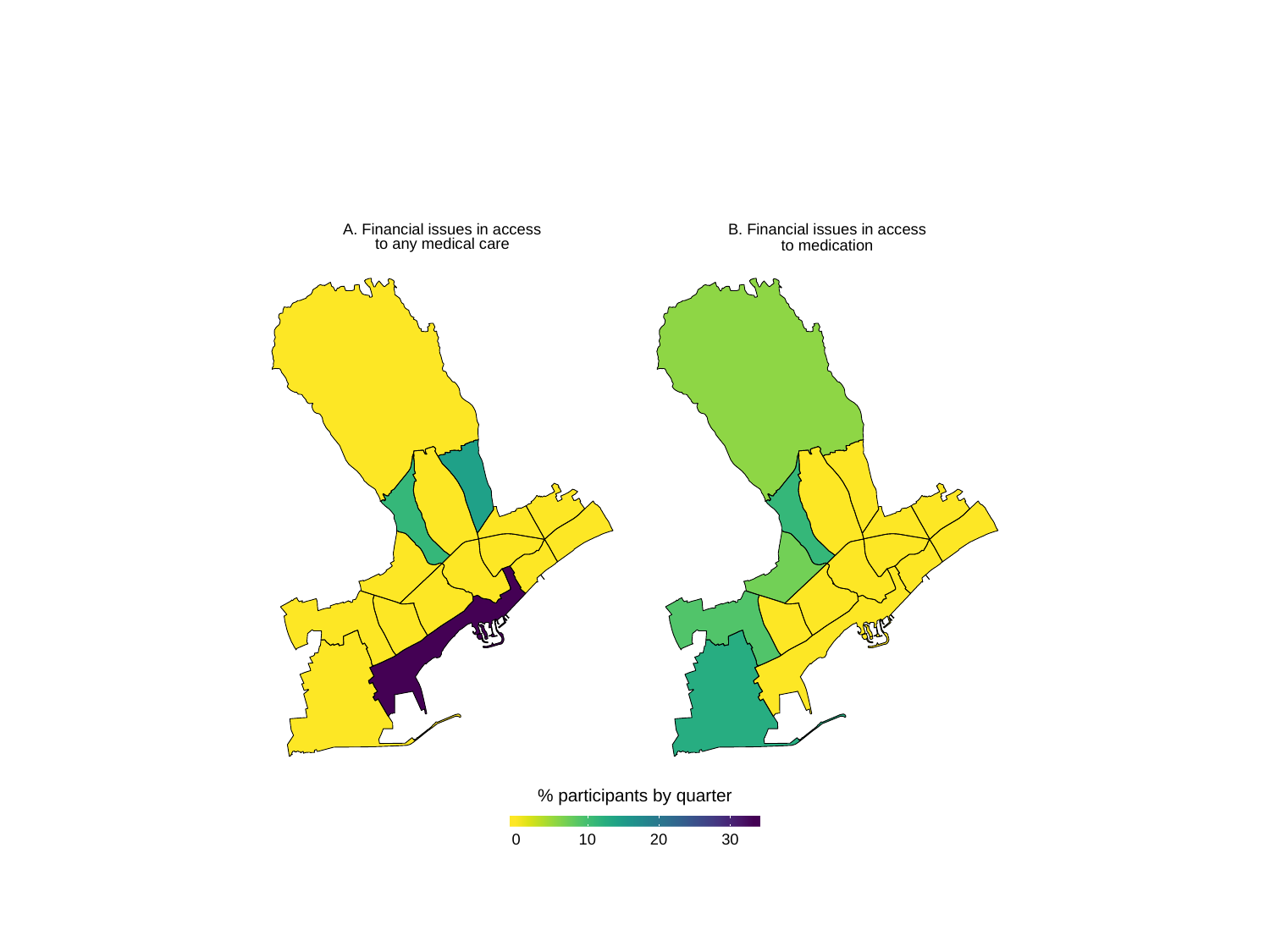

A. Financial issues in access
B. Financial issues in access
to any medical care
to medication
% participants by quarter
30
0
10
20

## Slide 7
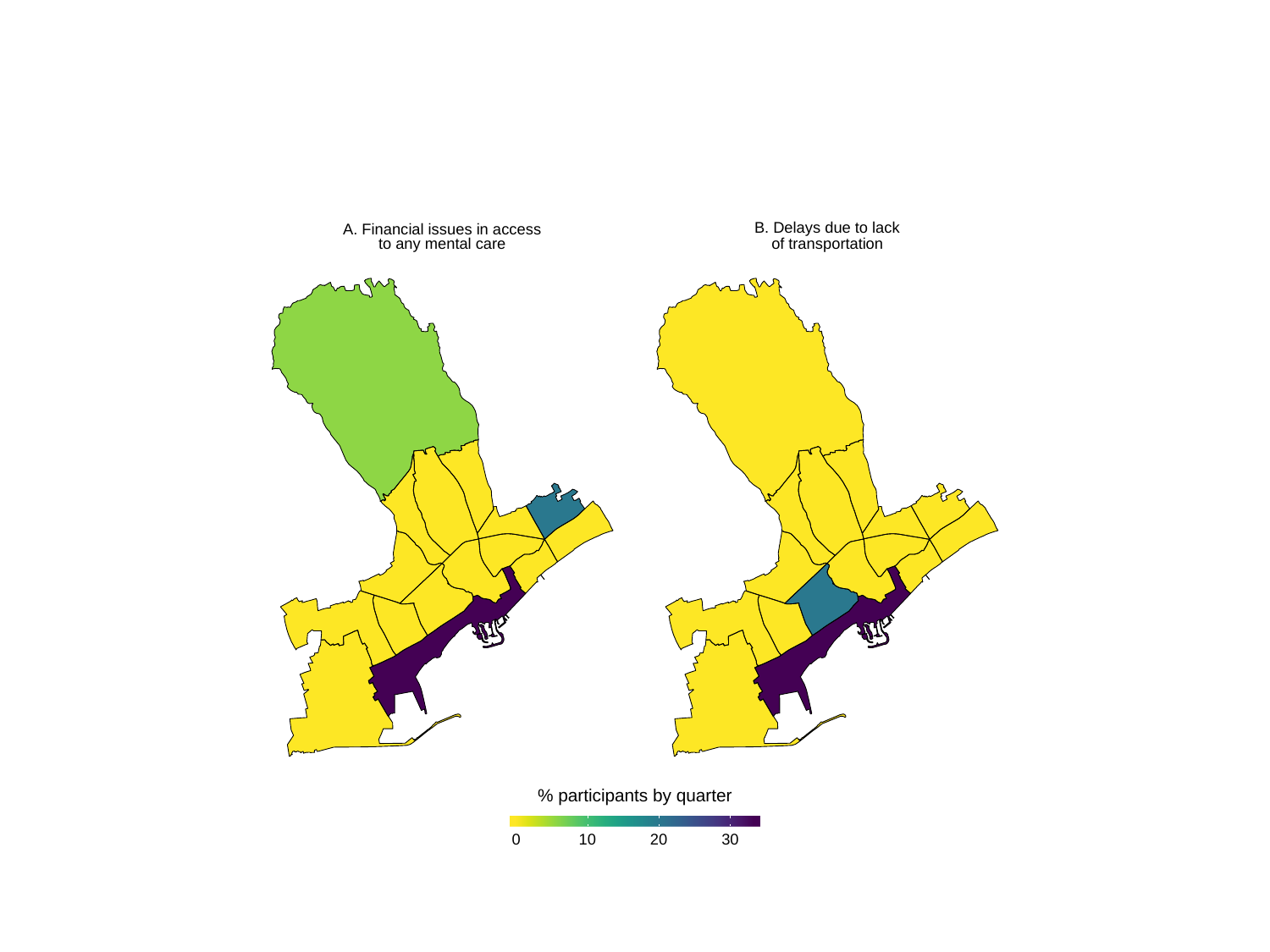

B. Delays due to lack
A. Financial issues in access
of transportation
to any mental care
% participants by quarter
30
0
10
20

## Slide 8
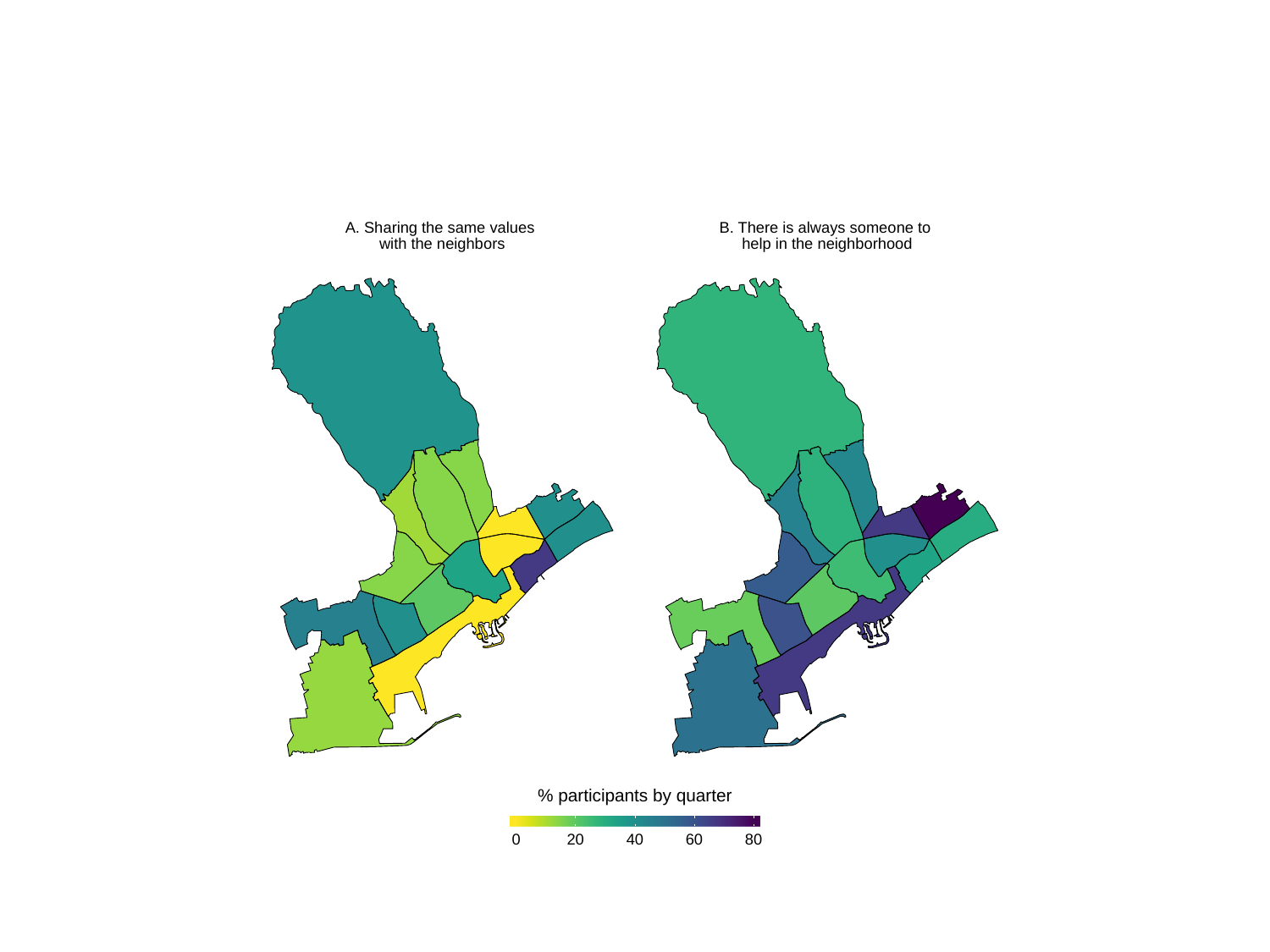

A. Sharing the same values
B. There is always someone to
with the neighbors
help in the neighborhood
% participants by quarter
0
20
40
60
80

## Slide 9
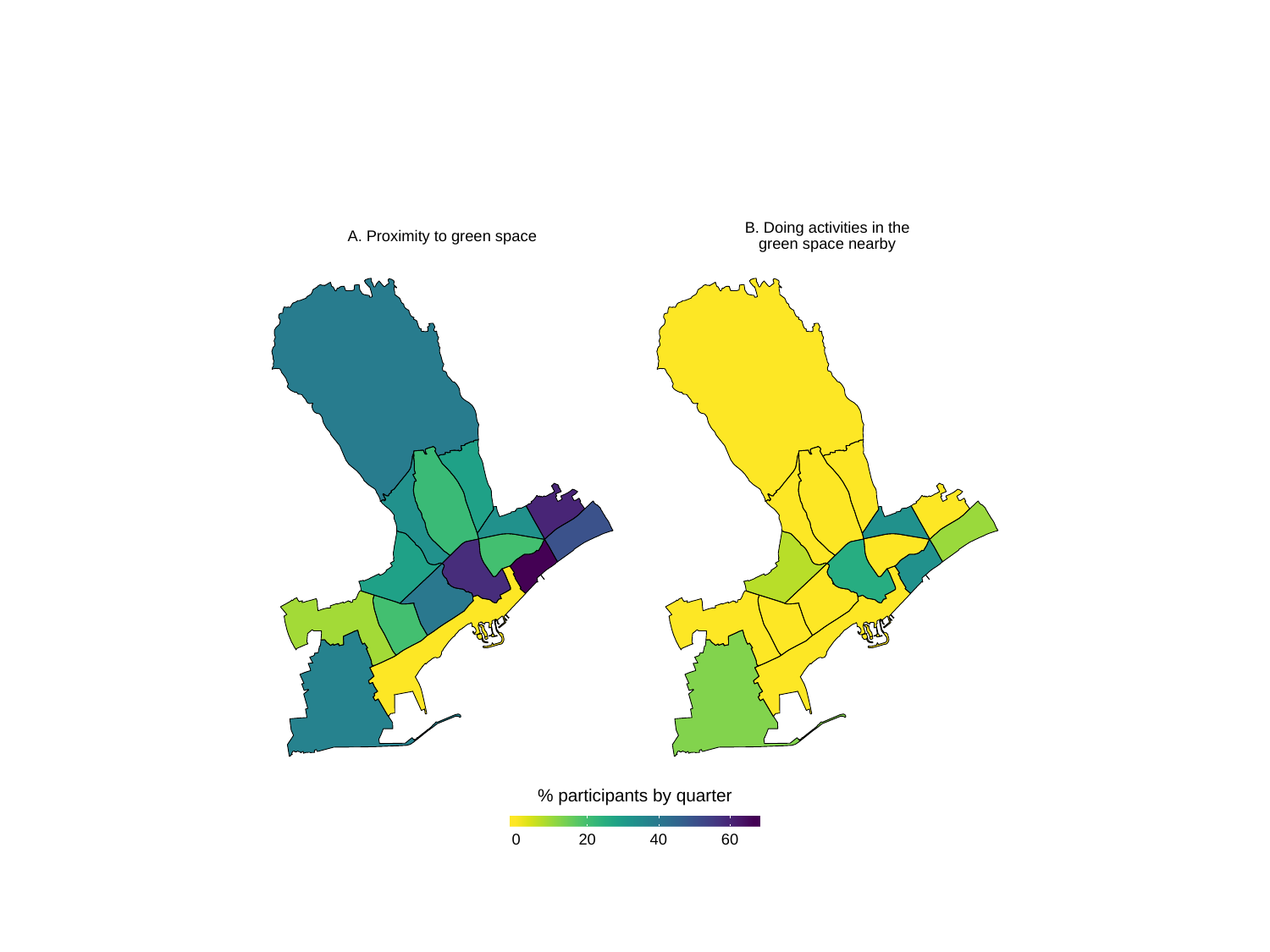

B. Doing activities in the
A. Proximity to green space
green space nearby
% participants by quarter
0
20
40
60

## Slide 10
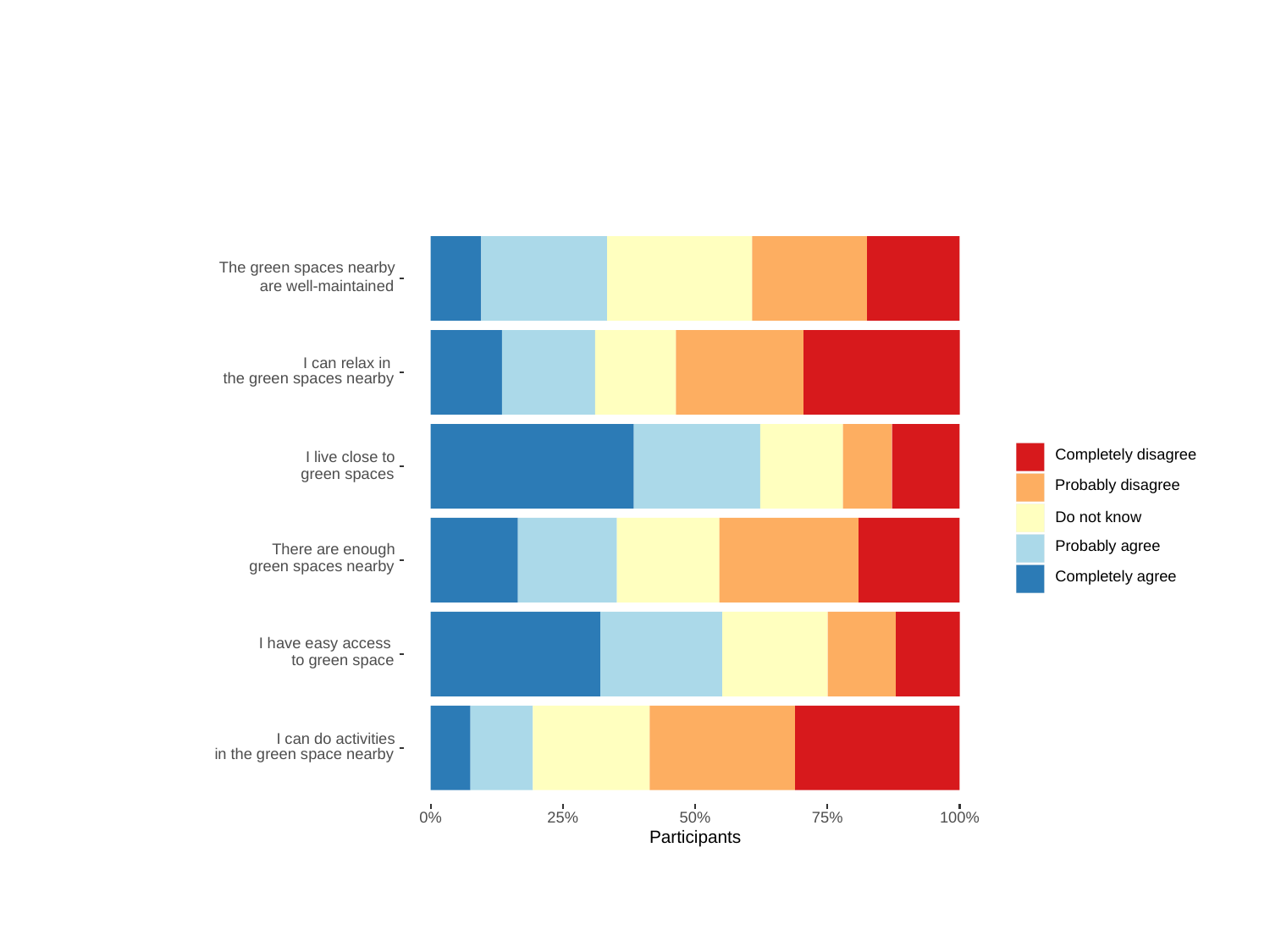

The green spaces nearby
 are well-maintained
I can relax in
 the green spaces nearby
Completely disagree
I live close to
 green spaces
Probably disagree
Do not know
Probably agree
There are enough
 green spaces nearby
Completely agree
I have easy access
 to green space
I can do activities
 in the green space nearby
0%
25%
50%
75%
100%
Participants

## Slide 11
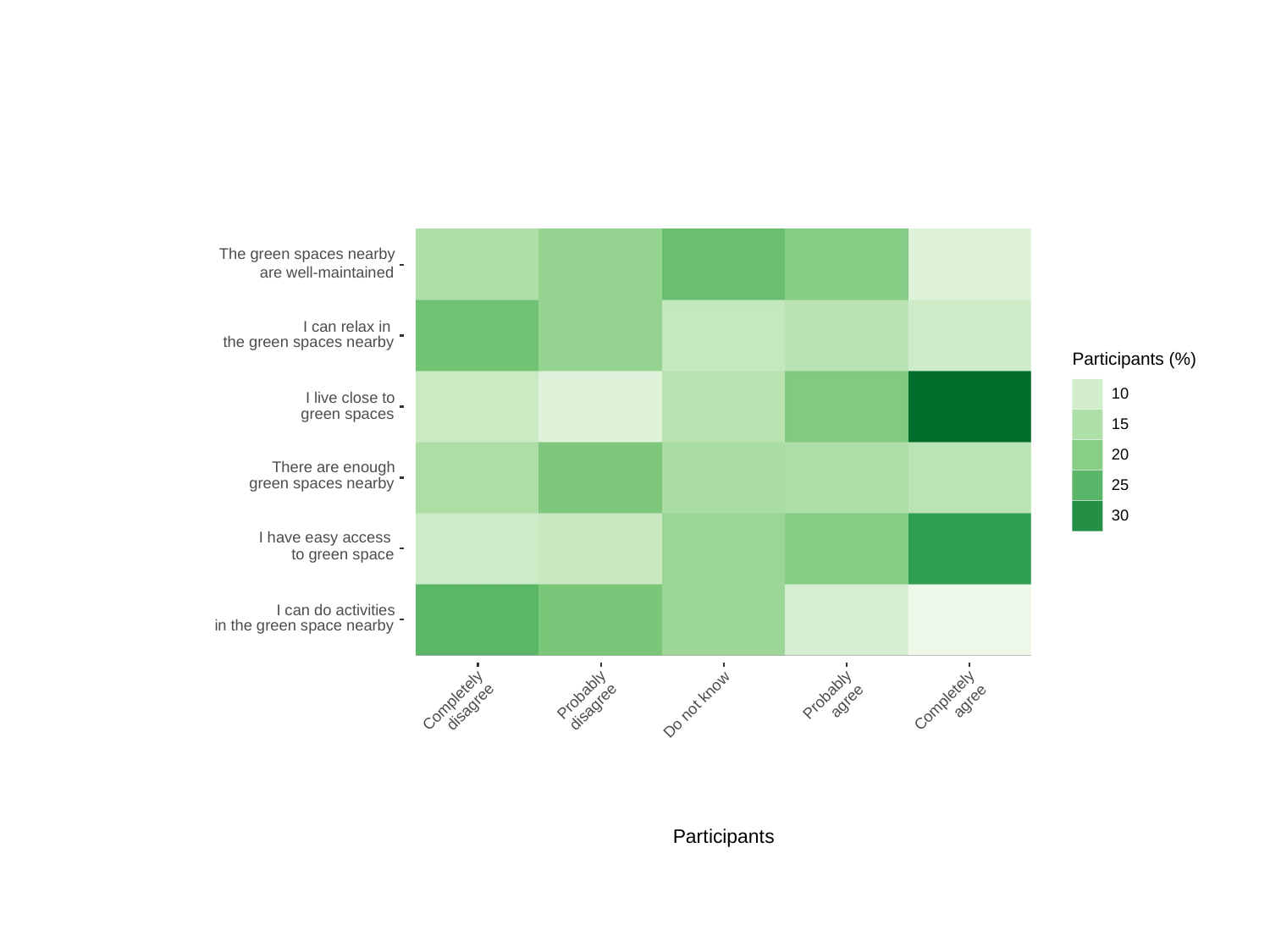

The green spaces nearby
 are well-maintained
I can relax in
 the green spaces nearby
Participants (%)
10
I live close to
 green spaces
15
20
There are enough
 green spaces nearby
25
30
I have easy access
 to green space
I can do activities
 in the green space nearby
Probably
Probably
Completely
Completely
Do not know
 agree
 agree
 disagree
 disagree
Participants

## Slide 12
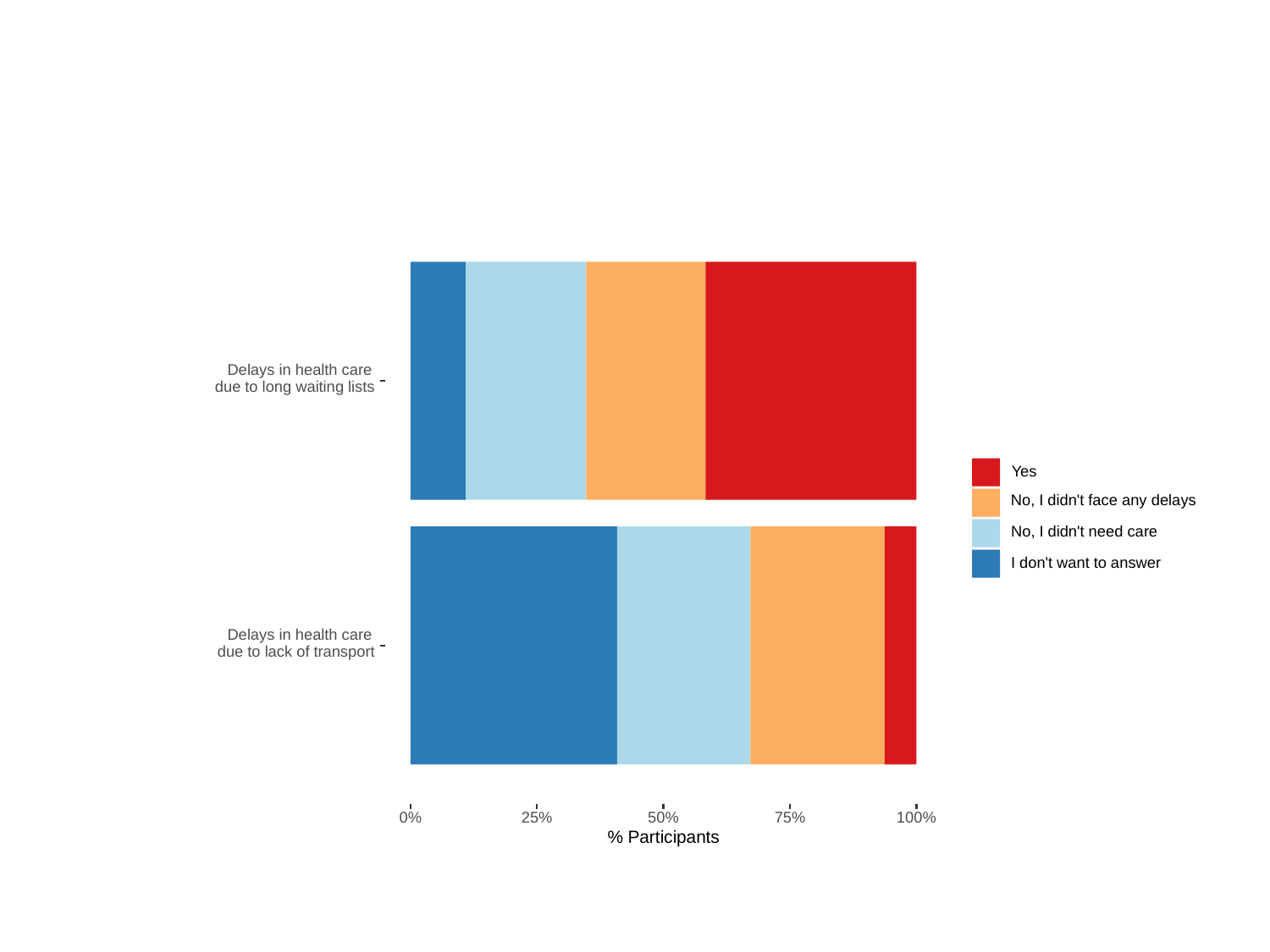

Delays in health care
 due to long waiting lists
Yes
No, I didn't face any delays
No, I didn't need care
I don't want to answer
Delays in health care
 due to lack of transport
0%
25%
50%
75%
100%
% Participants

## Slide 13
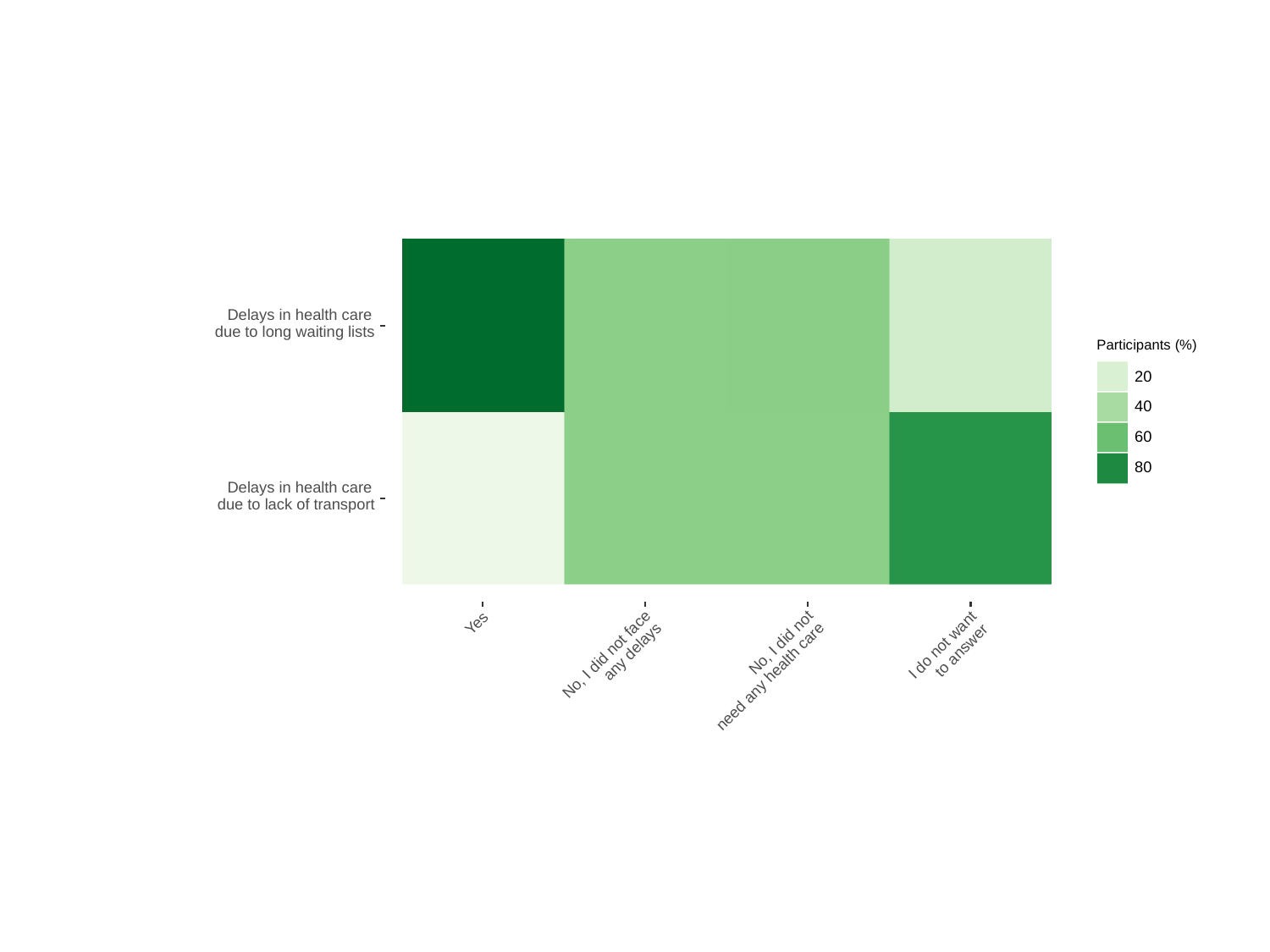

Delays in health care
 due to long waiting lists
Participants (%)
20
40
60
80
Delays in health care
 due to lack of transport
Yes
No, I did not
I do not want
No, I did not face
 any delays
 to answer
 need any health care

## Slide 14
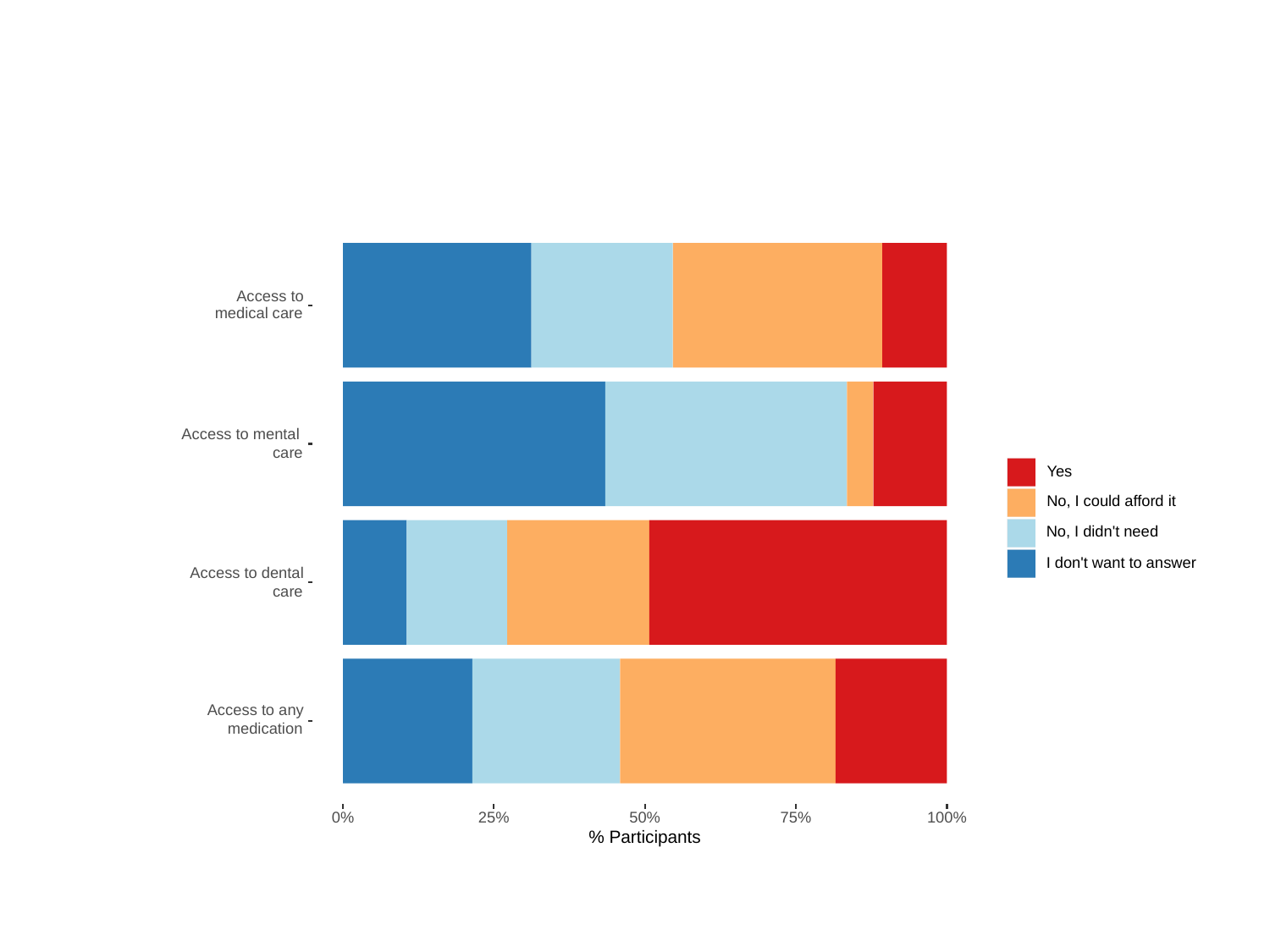

Access to
 medical care
Access to mental
 care
Yes
No, I could afford it
No, I didn't need
I don't want to answer
Access to dental
 care
Access to any
 medication
0%
25%
50%
75%
100%
% Participants

## Slide 15
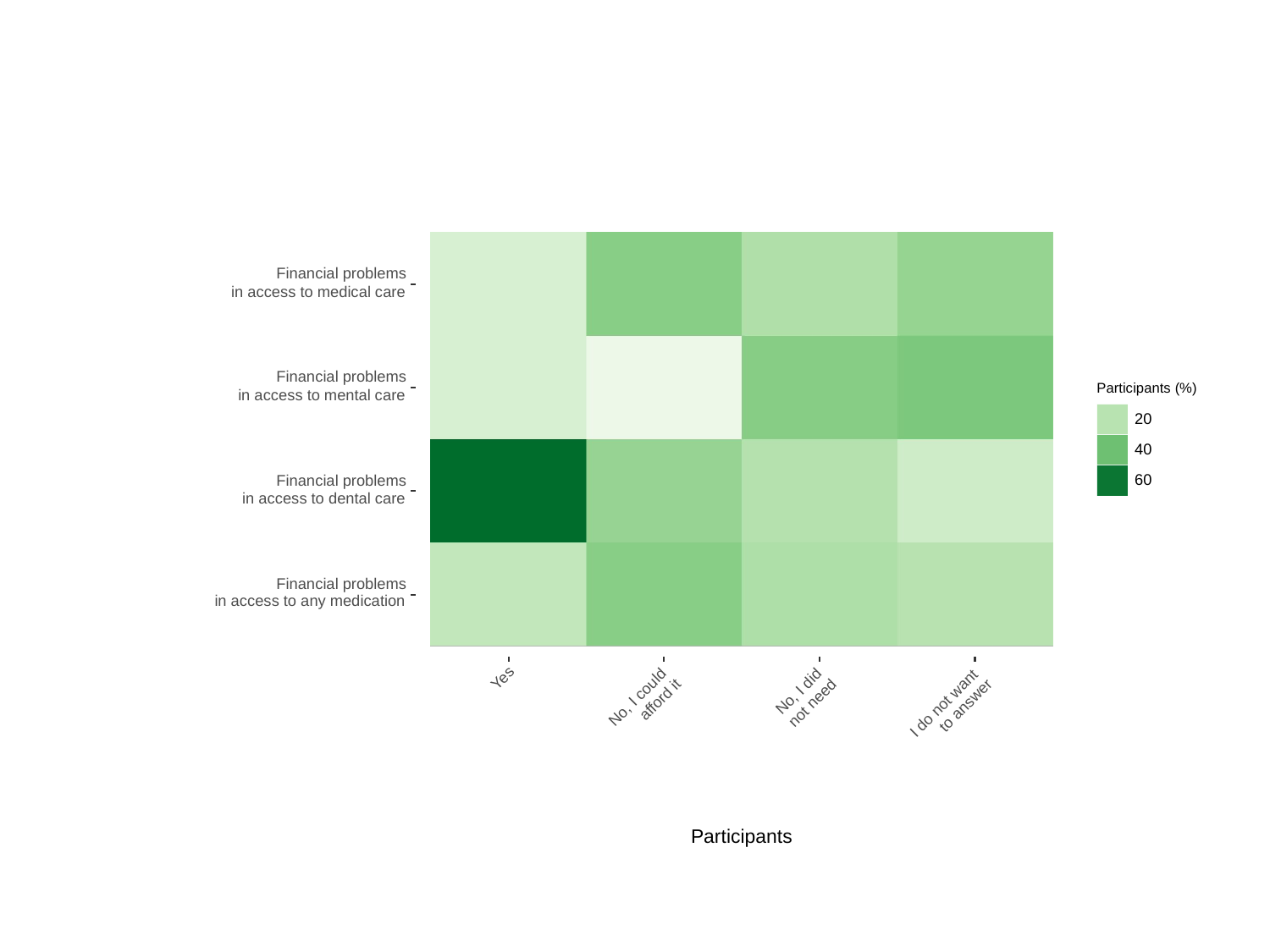

Financial problems
 in access to medical care
Financial problems
Participants (%)
 in access to mental care
20
40
Financial problems
60
 in access to dental care
Financial problems
 in access to any medication
Yes
No, I did
No, I could
I do not want
 afford it
 not need
 to answer
Participants

## Slide 16
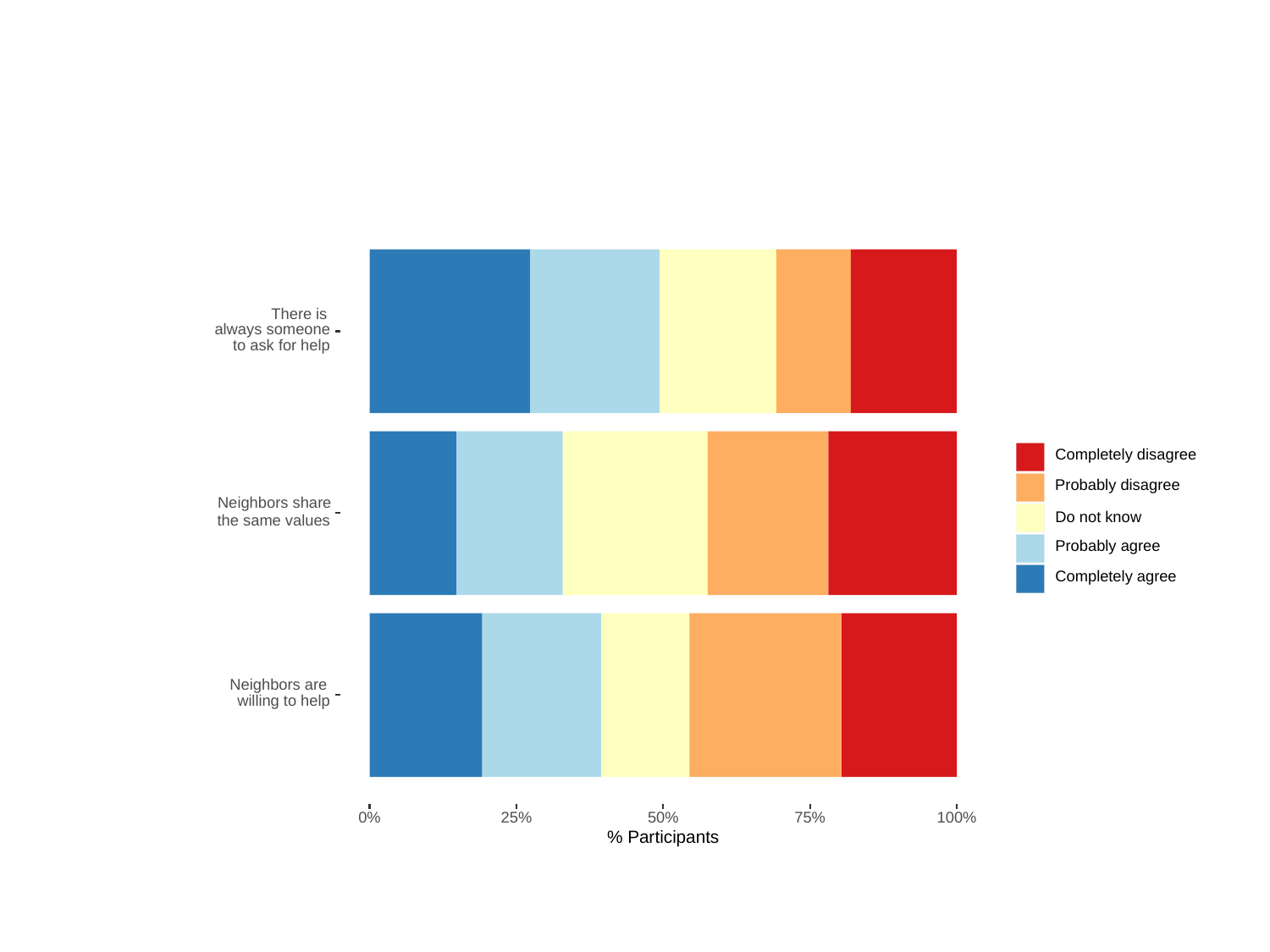

There is
 always someone
 to ask for help
Completely disagree
Probably disagree
Neighbors share
Do not know
 the same values
Probably agree
Completely agree
Neighbors are
 willing to help
0%
25%
50%
75%
100%
% Participants

## Slide 17
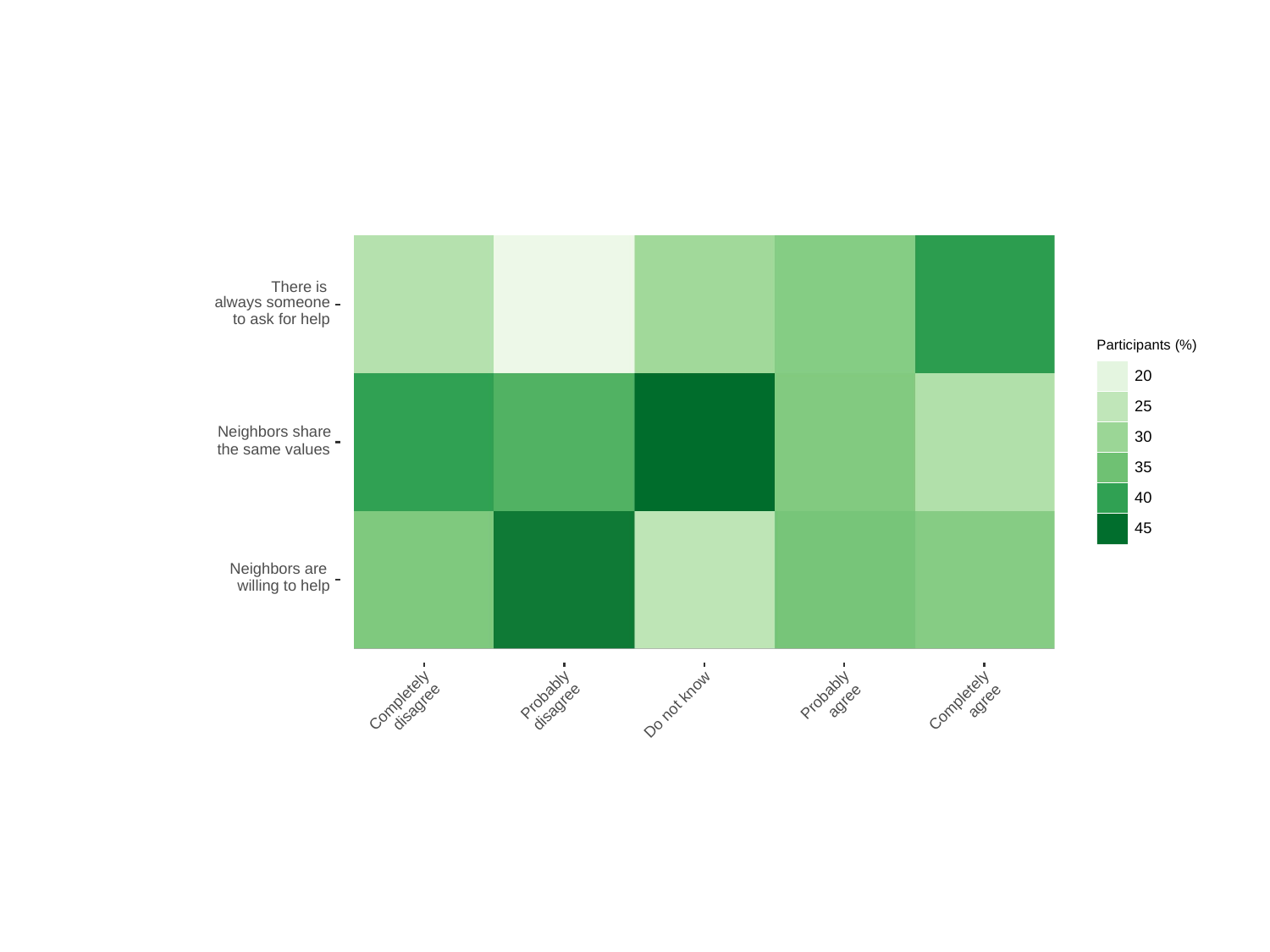

There is
 always someone
 to ask for help
Participants (%)
20
25
Neighbors share
30
 the same values
35
40
45
Neighbors are
 willing to help
Probably
Probably
Completely
Completely
Do not know
 agree
 agree
 disagree
 disagree

## Slide 18
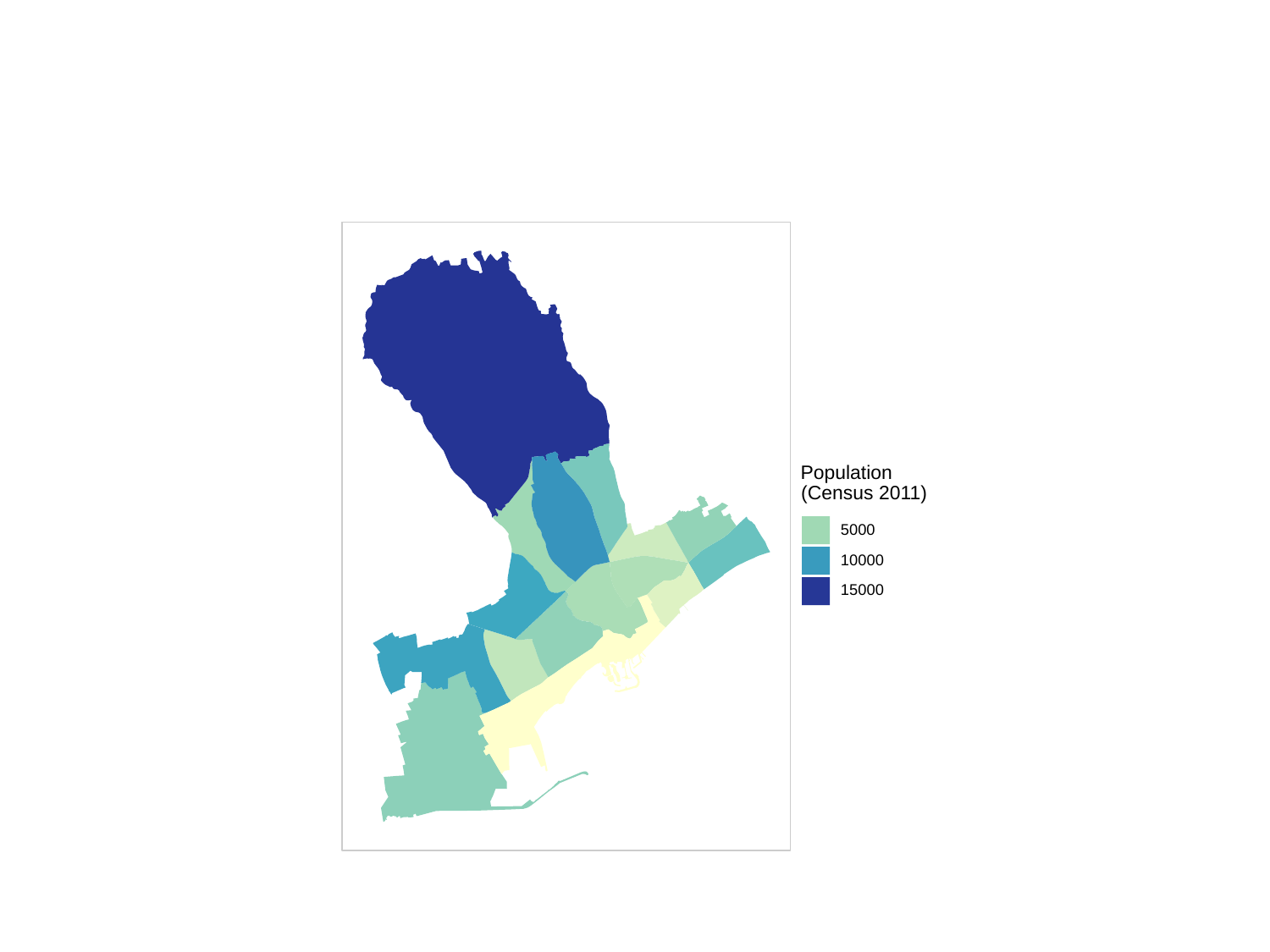

Population
(Census 2011)
5000
10000
15000
