# Supplementary material for: Application of the urban exposome framework using drinking water and quality of life indicators: a proof-of-concept study in Limassol, Cyprus
Source: PeerJ. 2019 May 24;7:e6851. doi: 10.7717/peerj.6851 (PMC6536114; doi:10.7717/peerj.6851)
Supplement: Supplemental Information 9 [file peerj-07-6851-s009.zip › SupplementalData_UrbanExposomeWater_PeerJ/UrbanExposomeWater_EWAS_2019-05-03.pptx]

## Slide 1
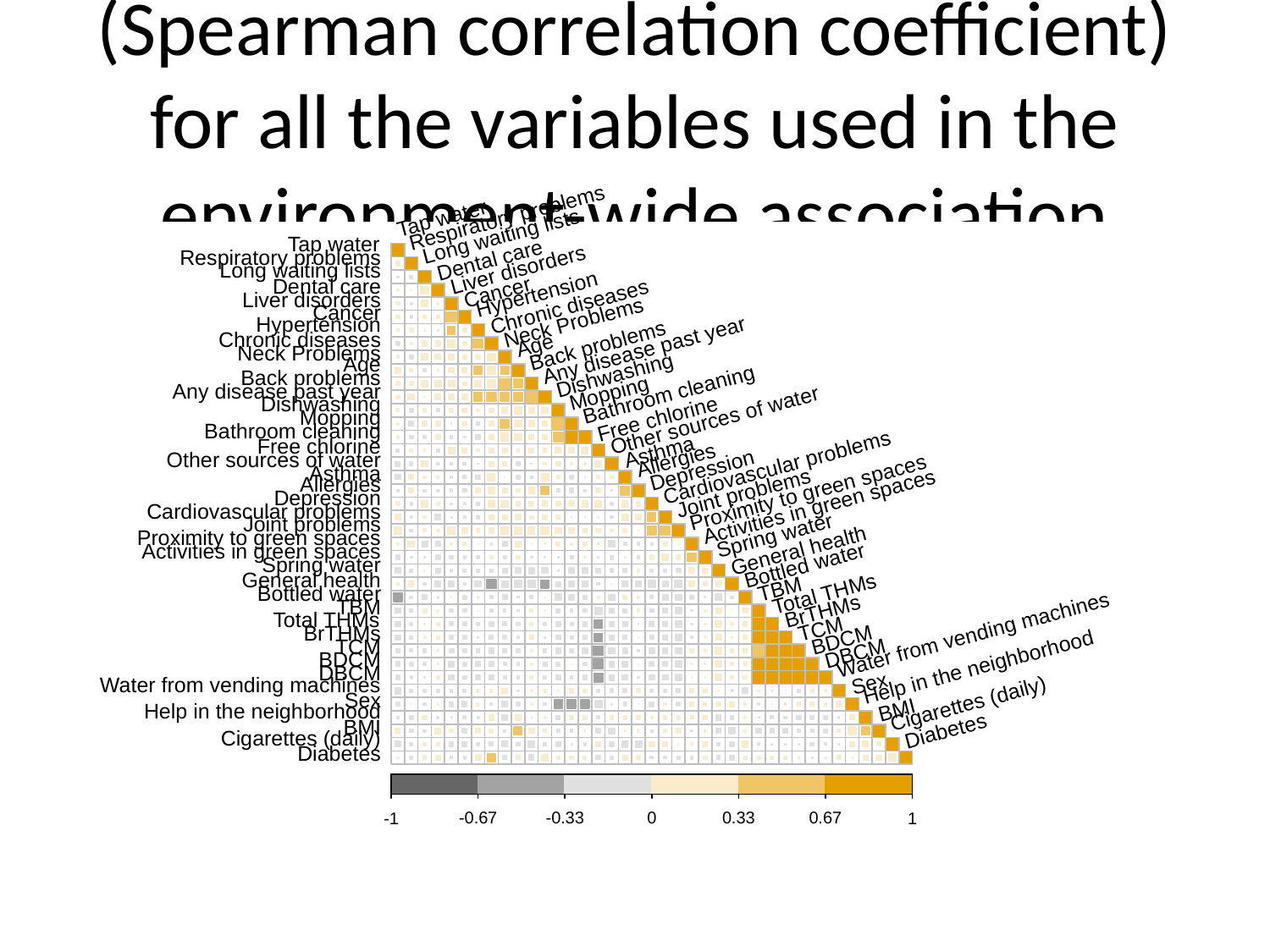

# Figure 7: Correlation plot (Spearman correlation coefficient) for all the variables used in the environment-wide association exploratory analysis.
Respiratory problems
Tap water
Long waiting lists
Tap water
Respiratory problems
Dental care
Long waiting lists
Liver disorders
Dental care
Cancer
Hypertension
Liver disorders
Chronic diseases
Cancer
Hypertension
Neck Problems
Chronic diseases
Age
Back problems
Any disease past year
Neck Problems
Age
Dishwashing
Back problems
Any disease past year
Mopping
Bathroom cleaning
Dishwashing
Mopping
Free chlorine
Other sources of water
Bathroom cleaning
Free chlorine
Asthma
Allergies
Other sources of water
Cardiovascular problems
Depression
Asthma
Allergies
Proximity to green spaces
Joint problems
Depression
Activities in green spaces
Cardiovascular problems
Joint problems
Spring water
Proximity to green spaces
Activities in green spaces
General health
Spring water
Bottled water
General health
TBM
Bottled water
Total THMs
TBM
BrTHMs
Total THMs
TCM
Water from vending machines
BrTHMs
BDCM
TCM
DBCM
BDCM
Help in the neighborhood
DBCM
Water from vending machines
Sex
Sex
Cigarettes (daily)
Help in the neighborhood
BMI
BMI
Diabetes
Cigarettes (daily)
Diabetes
-0.33
0.33
-0.67
0
0.67
-1
1

## Slide 2
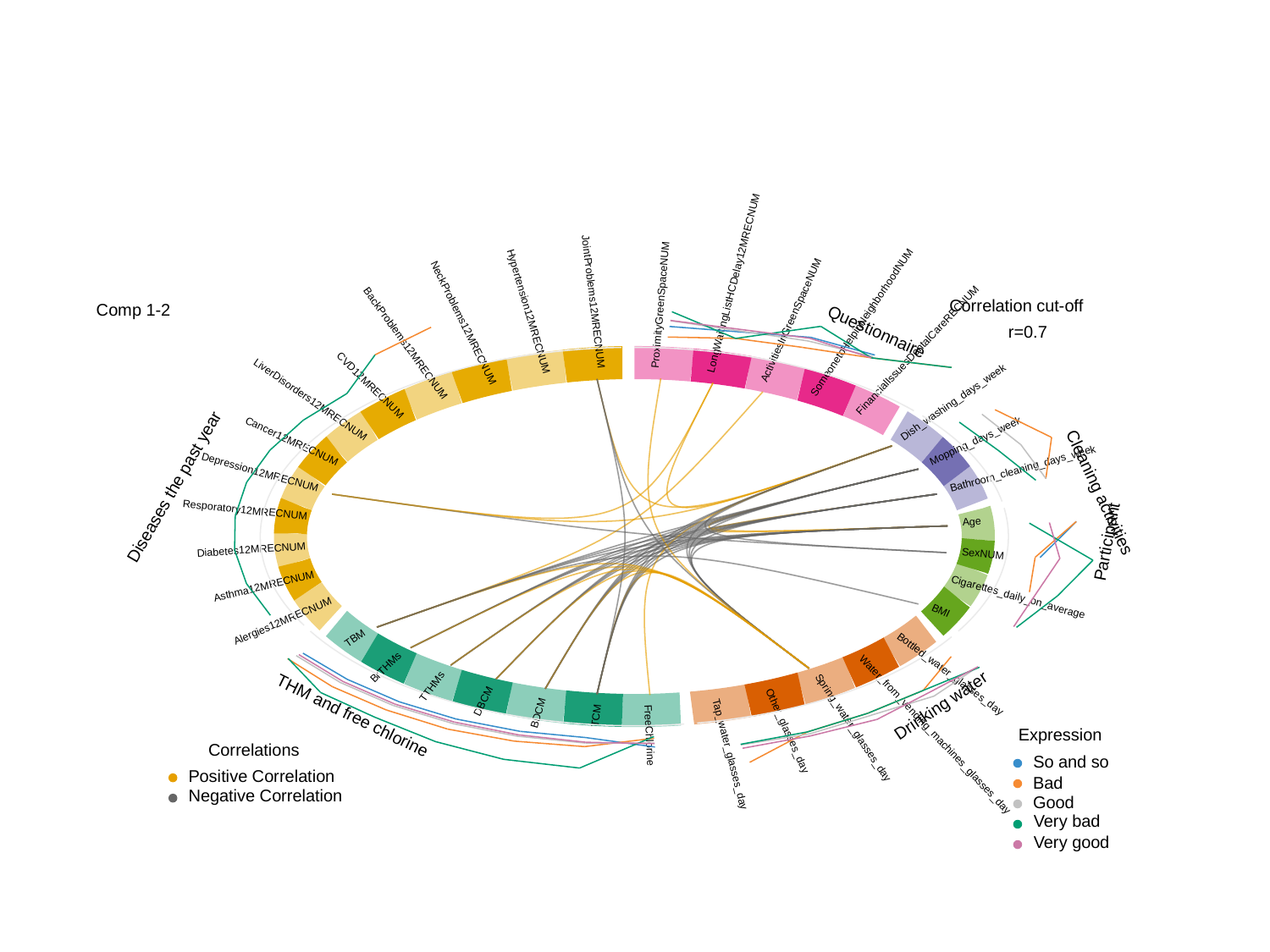

LongWaitingListHCDelay12MRECNUM
JointProblems12MRECNUM
ProximityGreenSpaceNUM
Correlation cut-off
Comp 1-2
Hypertension12MRECNUM
ActivitiesInGreenSpaceNUM
SomeonetoHelpinNeighborhoodNUM
NeckProblems12MRECNUM
Questionnaire
r=0.7
BackProblems12MRECNUM
FinancialIssuesDentalCareRECNUM
CVD12MRECNUM
LiverDisorders12MRECNUM
Dish_washing_days_week
Mopping_days_week
Cancer12MRECNUM
Bathroom_cleaning_days_week
Depression12MRECNUM
Diseases the past year
Cleaning activities
Resporatory12MRECNUM
Age
Participant
Diabetes12MRECNUM
SexNUM
Asthma12MRECNUM
Cigarettes_daily_on_average
BMI
Alergies12MRECNUM
TBM
BrTHMs
Bottled_water_glasses_day
TTHMs
DBCM
Drinking water
BDCM
THM and free chlorine
TCM
Spring_water_glasses_day
Other_glasses_day
Expression
Water_from_vending_machines_glasses_day
FreeChlorine
Correlations
Tap_water_glasses_day
So and so
Positive Correlation
Bad
Negative Correlation
Good
Very bad
Very good

## Slide 3
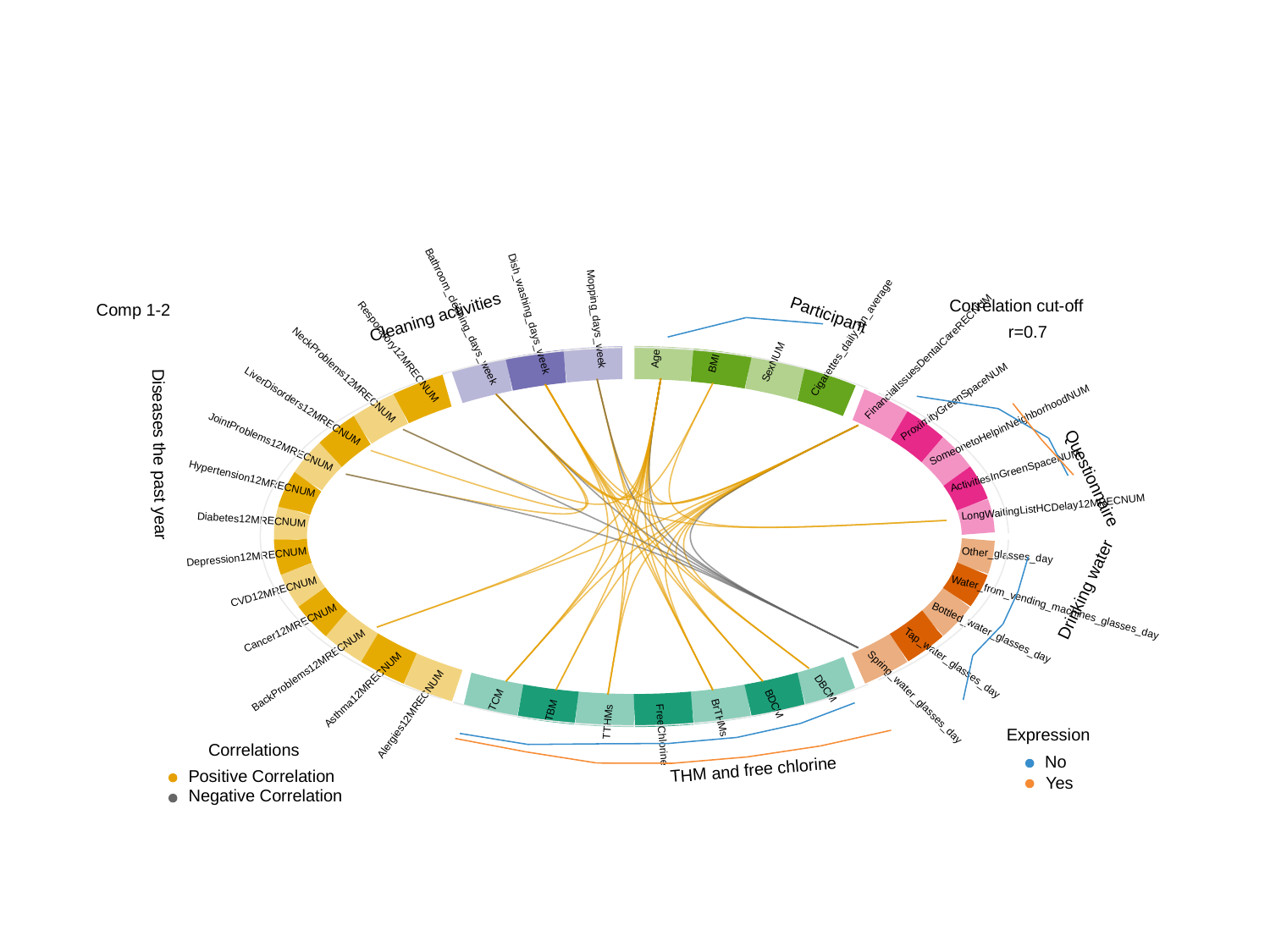

Correlation cut-off
Comp 1-2
Participant
Dish_washing_days_week
Cleaning activities
Bathroom_cleaning_days_week
Mopping_days_week
r=0.7
Cigarettes_daily_on_average
Resporatory12MRECNUM
FinancialIssuesDentalCareRECNUM
Age
SexNUM
BMI
NeckProblems12MRECNUM
ProximityGreenSpaceNUM
LiverDisorders12MRECNUM
SomeonetoHelpinNeighborhoodNUM
JointProblems12MRECNUM
Diseases the past year
ActivitiesInGreenSpaceNUM
Questionnaire
Hypertension12MRECNUM
LongWaitingListHCDelay12MRECNUM
Diabetes12MRECNUM
Other_glasses_day
Depression12MRECNUM
Drinking water
CVD12MRECNUM
Water_from_vending_machines_glasses_day
Cancer12MRECNUM
Bottled_water_glasses_day
Tap_water_glasses_day
BackProblems12MRECNUM
DBCM
Asthma12MRECNUM
Spring_water_glasses_day
TCM
BDCM
TBM
Alergies12MRECNUM
BrTHMs
TTHMs
Expression
FreeChlorine
Correlations
No
THM and free chlorine
Positive Correlation
Yes
Negative Correlation
